# Characterizing college science instruction: The Three-Dimensional Learning Observation Protocol

**DOI:** 10.1371/journal.pone.0234640

**Published:** 2020-06-16

**Authors:** Kinsey Bain, Lydia Bender, Paul Bergeron, Marcos D. Caballero, Justin H. Carmel, Erin M. Duffy, Diane Ebert-May, Cori L. Fata-Hartley, Deborah G. Herrington, James T. Laverty, Rebecca L. Matz, Paul C. Nelson, Lynmarie A. Posey, Jon R. Stoltzfus, Ryan L. Stowe, Ryan D. Sweeder, Stuart H. Tessmer, Sonia M. Underwood, Mark Urban-Lurain, Melanie M. Cooper

**Affiliations:** 1 Department of Chemistry, Michigan State University, East Lansing, Michigan, United States of America; 2 Department of Physics, Kansas State University, Manhattan, Kansas, United States of America; 3 Department of Physics and Astronomy, Michigan State University, East Lansing, Michigan, United States of America; 4 CREATE for STEM Institute, Michigan State University, East Lansing, Michigan, United States of America; 5 Department of Physics and Center for Computing in Science Education, University of Oslo, Oslo, Norway; 6 Department of Chemistry & Biochemistry and STEM Transformation Institute, Florida International University, Miami, Florida, United States of America; 7 Department of Chemistry & Science, Math, and Technology Education, Western Washington University, Bellingham, Washington, United States of America; 8 Department of Plant Biology, Michigan State University, East Lansing, Michigan, United States of America; 9 College of Natural Science, Michigan State University, East Lansing, Michigan, United States of America; 10 Department of Chemistry, Grand Valley State University, Allendale, Michigan, United States of America; 11 Hub for Innovation in Learning and Technology, Michigan State University, East Lansing, Michigan, United States of America; 12 Department of Biochemistry and Molecular Biology, Michigan State University, East Lansing, Michigan, United States of America; 13 Department of Chemistry, University of Wisconsin-Madison, Madison, Wisconsin, United States of America; 14 Lyman Briggs College, Michigan State University, East Lansing, Michigan, United States of America; Florida Agricultural and Mechanical University, UNITED STATES

## Abstract

The importance of improving STEM education is of perennial interest, and to this end, the education community needs ways to characterize transformation efforts. Three-dimensional learning (3DL) is one such approach to transformation, in which core ideas of the discipline, scientific practices, and crosscutting concepts are combined to support student development of disciplinary expertise. We have previously reported on an approach to the characterization of assessments, the Three-Dimensional Learning Assessment Protocol (3D-LAP), that can be used to identify whether assessments have the potential to engage students in 3DL. Here we present the development of a companion, the Three-Dimensional Learning Observation Protocol (3D-LOP), an observation protocol that can reliably distinguish between instruction that has potential for engagement with 3DL and instruction that does not. The 3D-LOP goes beyond other observation protocols, because it is intended not only to characterize the pedagogical approaches being used in the instructional environment, but also to identify whether students are being asked to engage with scientific practices, core ideas, and crosscutting concepts. We demonstrate herein that the 3D-LOP can be used reliably to code for the presence of 3DL; further, we present data that show the utility of the 3D-LOP in differentiating between instruction that has the potential to promote 3DL from instruction that does not. Our team plans to continue using this protocol to evaluate outcomes of instructional transformation projects. We also propose that the 3D-LOP can be used to support practitioners in developing curricular materials and selecting instructional strategies to promote engagement in three-dimensional instruction.

## Introduction

Science is advancing at a breakneck pace. With discoveries like gravitational waves [1], the use of CRISPR technology to treat sickle cell disease [2], and self-healing polymers [3] in just the past couple of years, science education that prepares students for careers in current science fields–and those of tomorrow–and promotes scientific literacy is essential. Discipline-based education research (DBER) has clearly shown that research-based pedagogical strategies can positively influence student learning outcomes as compared to traditional lecture-based approaches, although evidence about the relative effectiveness of specific strategies on learning is limited [4]. Despite this, changes in science education, particularly in introductory-level college science courses, have not led to the concomitant, widespread, and robust transformation that would be expected to support students as they navigate this rapidly changing landscape [5,6]. In many ways, science education largely looks the same today as it did 70 years ago.

To date, much research involving pedagogical approaches has coalesced around what is now known as “active learning”–the idea that students must be actively engaged with their learning [5,7,8]. Until recently, however, what students should learn and what they should do with that knowledge has not received as much attention. This issue is of particular importance because while these active learning studies show promise, there remains ample evidence that students leave science courses and even degree programs with incomplete or incorrect understanding of important disciplinary ideas and an inability to transfer knowledge to new situations [4,9]. Such evidence reveals that there is still much work to do to help students develop coherent and interconnected knowledge that can be used in a variety of situations. Shifting the focus away from how classes are taught to what is being taught, an idea rooted in ample literature such as pedagogical content knowledge, would allow for concentrated study on the interactive time that comprises students’ learning experiences in undergraduate science courses [10,11].

Here, we report on our continuing work to transform gateway science courses by moving beyond active learning to incorporate what has become known as three-dimensional learning (3DL) which originates from *A Framework for K-12 Science Education* (the Framework), a consensus report from the National Research Council [12]. Based on the best available research on student learning in the sciences, the Framework put forth a vision for science education in which curricula would be restructured as scaffolded progressions for each of the three dimensions: disciplinary core ideas (fundamental concepts that underpin a discipline), scientific and engineering practices (what scientists do with their knowledge), and crosscutting concepts (tools or lenses used across disciplines for making sense of phenomena) [9,12–16].

The three dimensions are used in concert by practicing scientists and engineers when applying their knowledge to investigate and reason about phenomena; therefore, the Framework stresses that the dimensions should be integrated throughout curricula, instruction, and assessment to support moving students toward more expert-like engagement in science over time. The impetus behind 3DL is a response to the status quo of traditional science learning environments, where instruction and assessment typically focus on collections of facts and skills [17,18]. In these settings, it is common for content and skills to be disaggregated, resulting in fragmented knowledge for learners [4,9,13,19]. In contrast, 3DL is designed to promote the development and use of interconnected knowledge that is more expert-like in nature [9,12–16,19,20]. Such knowledge is contextualized, connected, and useful, rather than a collection of facts, ideas, and calculations that are disconnected from scientific ideas. Although written for a K-12 context, the Framework’s vision can and should be used as a guide to restructuring science curricula in higher education [9,13–16,20,21]. In addition, implementing 3DL in college science courses provides opportunities to build coherently upon students’ prior science learning.

To support the adaptation of 3DL to science courses at the college level, our team has developed two protocols that characterize the extent to which assessments and instruction in gateway biology, chemistry, and physics courses provide opportunities for students to engage with the three dimensions. The previously published Three-Dimensional Learning Assessment Protocol (3D-LAP) is useful as a tool for both research and faculty professional development [13]. For example, we have used the 3D-LAP to track changes in assessment practice over time in gateway science courses as one measure of course transformation [16], as well as to explicitly guide instructors in writing and adapting assessments that can engage students with 3DL [20]. Providing such support to practitioners is critical to propagating course transformation beyond the research team. The Three-Dimensional Learning Observation Protocol (3D-LOP), reported here, was developed to characterize instruction in gateway biology, chemistry, and physics courses for both evidence-based pedagogical practices and the potential for engagement with the three dimensions. The 3D-LOP can be used to evaluate courses and support research on transformation efforts. In addition, the 3D-LOP can be used as a professional development tool for faculty as it provides feedback to support the development and modification of instructional practice and materials.

### Focusing on instruction and characterizing instructional practices

In recent years there has been an increased focus on instruction in undergraduate science, technology, engineering, and mathematics (STEM) disciplines, often centered on promoting the adoption and implementation of more student-centered pedagogies, which have become familiar under the banner of “active learning” [4,5,7,8]. The research on active learning generally falls into two categories: 1) demonstrating impact on students as shown either by changes in course grades or responses to concept inventories, and 2) characterizing learning environments using observation protocols.

Teaching strategies that support student engagement can lead to improved student performance and retention in STEM courses compared to outcomes for students in traditional course settings [7,8]. However, active learning can refer to a host of instructional approaches, from the adoption of clicker-based technology to answer multiple-choice questions in lecture courses, to “flipped classrooms” where some material is presented outside of class allowing students to spend more class time engaged in some activity, to studio courses where students work in groups on open-ended problems [5,7,8]. Despite this lack of clarity about the meaning of active learning, calls to incorporate active learning have strengthened over the past ten years [4,5,7–9,15,16,22]. Some evidence of the positive impacts of active learning indicates that students who are struggling are most likely to benefit from these instructional strategies. For example, Freeman et al. showed that compared to their traditional counterparts, students in active learning courses had lower DFW rates (that is, students receiving a grade of D or F and students withdrawing from the course) and higher scores on exams and concept inventories [7].

Despite numerous calls to actively engage students during class sessions in STEM courses, the recent large-scale study by Stains et al. shows that instructor-centered practices still prevail in undergraduate STEM courses when characterized with the Classroom Observation Protocol for Undergraduate STEM (COPUS) [5,23]. Other comparable observation protocols, such as the Real-time Instructor Observing Tool (RIOT), Reformed Teaching Observation Protocol (RTOP), and Teaching Dimensions Observation Protocol (TDOP), have been developed and used for similar purposes [24–26]. These protocols are limited, however, in that they focus on *how* a class is being taught without providing information on *what* is being taught and what students are expected to do with their knowledge. Because these protocols focus on instructor and student activities (e.g., working in groups, using clickers, or asking questions) or classroom environments (e.g., climate, reflective practices, or participation), they are best suited for characterizing instruction on the instructor-centered to student-centered spectrum. While this information can provide a rich characterization of the learning environment, it is possible that an engaged classroom as characterized by one such protocol may engage students only in developing rote skills, completing algorithmic calculations, or recalling facts. Further, undergraduate and even graduate students often finish STEM courses and complete degrees without a firm grasp of foundational ideas and sufficient practice in transferring knowledge to new contexts [4,27]. Even students who have taken courses that incorporate active learning often have yet to construct the necessary resources and interconnections needed to effectively use important science ideas [28].

We propose that it is time to move beyond “active learning” and focus on both *how* science is taught (i.e., what instructors and students are doing) and *what* is taught (i.e., what students should know and be able to do) in undergraduate courses by incorporating 3DL in college science instruction and assessment [13]. Moreover, 3DL is inherently active because it requires students to explore core ideas through engagement with science and engineering practices and the application of crosscutting concepts [16]. Indeed, if we transform courses according to the Framework and implement assessments that align with this vision, what happens in the classroom must also change to maintain alignment; effective instruction must afford students the opportunity to gain experience in engaging with each of the three dimensions together in formative situations before summative assessment. Our goal was to develop the 3D-LOP as a tool that can characterize the extent to which instruction (the learning environment, instructional techniques, content, and how that content is used) has the potential to engage students with the three dimensions and the nature of that engagement (student-centered vs. instructor-centered).

The 3D-LOP complements the 3D-LAP; together they can be used to comprehensively characterize course transformations and support faculty professional development. Here, we describe the development of the 3D-LOP and provide evidence that the protocol can distinguish between instruction that has the potential to engage students with the three dimensions outlined in the Framework and instruction that does not.

## Methods

### The Three-Dimensional Learning Observation Protocol (3D-LOP)

The purpose of the 3D-LOP is to characterize instruction in college-level biology, chemistry, and physics courses; therefore, the coding criteria target opportunities provided through instruction for students to engage with each of the dimensions in these three disciplines. The 3D-LOP was not designed to determine the degree to which students engage with the curricular materials, but rather whether instruction *offers opportunities* to engage in 3DL. As with the 3D-LAP, development of the 3D-LOP began with a review of the Framework. Further, because the 3D-LAP was completed first, it greatly influenced the development of the 3D-LOP. The research team, composed of disciplinary experts (many who identify as discipline-based education researchers) in biology, chemistry, and physics, used similar adaptations of the Framework for both the 3D-LAP and 3D-LOP.

Core ideas are explanatory and generative concepts fundamental to a discipline that underlie commonly taught topics in that discipline. The specific core ideas described in the 3D-LAP and 3D-LOP (Table 1) purposefully differ from those in the Framework for two reasons: the core ideas in the Framework are foundational but insufficient for college-level content, and they are organized for physical and biological sciences which is somewhat misaligned with the disciplinary organization of college-level science courses and degree programs. The core ideas for biology, chemistry, and physics used in the 3D-LAP and 3D-LOP were identified by members of the research team and colleagues from their respective departments [13,29]; it should be noted that these core ideas may not be suitable for all courses at all levels, and we recommend that faculty consider what core ideas they believe are appropriate. The scientific practices (Table 2) were used as described in the Framework with two exceptions: 1) engineering practices were not included because our focus was on developing a protocol for science courses and our collective expertise did not include engineering, and 2) constructing arguments and explanations were combined because the criteria for both are similar. The crosscutting concepts (Table 2) were also used as presented in the Framework with the exception that scale, proportion, and quantity was subdivided into two categories (Table 2).

**Table 1:** The core ideas for biology, chemistry, and physics in the 3D-LAP and 3D-LOP.

Biology Core Ideas	Chemistry Core Ideas	Physics Core Ideas
1. Chemical and physical basis of life 2. Matter and energy 3. Cellular basis of life 4. Systems 5. Structure and function 6. Information flow, exchange, and storage 7. Evolution	1. Electrostatic and bonding interactions 2. Atomic/molecular structure and properties 3. Energy: Macroscopic, atomic/molecular, quantum mechanical 4. Change and stability in chemical systems	1. Interactions can cause changes in motion 2. Energy is conserved 3. Exchanges of energy increase total entropy 4. Interactions are mediated by fields 5. Energy, momentum, angular momentum, and information can be transported without net transfer of matter

**Table 2:** The scientific practices and crosscutting concepts in the 3D-LAP and 3D-LOP.

Scientific Practices	Crosscutting Concepts
1. Asking questions 2. Developing and using models 3. Planning investigations 4. Analyzing and interpreting data 5. Using mathematics and computational thinking 6. Constructing explanations and engaging in argument from evidence 7. Evaluating information	1. Patterns 2. Cause and effect: Mechanism and explanation 3. Scale 4. Proportion and quantity 5. Systems and system models 6. Energy and matter: Flows, cycles, and conservation 7. Structure and function 8. Stability and change

The scientific practices and crosscutting concepts are adapted from the Framework and are applicable to biology, chemistry, and physics.

### Data collection

Video recordings of introductory biology, chemistry, and physics class sessions at Michigan State University (MSU) were collected during the first and third academic years of a three-year project [16]. Instructors from 65 (80%) of the 81 unique course sections opted into the study and allowed us to record their courses during this time. The recordings focused on the instructor’s actions, not on students, and instructors were assured that the recordings would be used only for programmatic improvement, not for individual evaluations. We aimed to record each course at the following three times: 1) toward the beginning of the semester (in weeks 3–6), 2) in the middle of the semester (in weeks 7–10), and 3) toward the end of the semester (in weeks 11–14). We asked instructors to avoid scheduling their recordings on review days or days where they were administering quizzes or exams. Other than that, the choice of which days to record was left to the discretion of the instructor(s) and the ability of our team to schedule someone to record the class meeting.

In total, we were able to collect 181 recordings across these 65 course sections, each ranging in length from approximately 50 to 80 minutes, the two standard class session lengths at MSU. Recordings were captured with a tripod-mounted video camera located in the back of the classroom. While the recordings were focused on the instructor and the course materials (board work, projected slides, demonstrations, etc.), interactions between the instructor and students were also noted. About 20% of the recordings from the total dataset (39 of 181) were used in the development of the 3D-LOP, selected to span all the disciplines, courses, and semesters of interest to help ensure that the final protocol would be robust for the entire dataset. Similarly, once the 3D-LOP criteria were finalized, 18 additional recordings (again spanning the disciplines, courses, and semesters of interest) were selected and coded to demonstrate reliability. The MSU Institutional Review Board deemed this project exempt in accordance with federal regulations. Instructors opted into this study and provided consent via email.

### Development of the 3D-LOP

#### Dividing each recording into segments

Some observation protocols define a fixed length of time with the coder recording all applicable activities that occur during each time segment (e.g., the COPUS [23] uses units of two minutes). However, coding recordings with fixed time units is not feasible for characterizing three-dimensional instruction where the focus is the nature of what students are expected to be learning, rather than what they are doing. We determined that a grain size larger than just a few minutes was typically needed to capture instructional arcs that engage students with a science practice (e.g., developing and using models or constructing explanations and engaging in argument from evidence). Disciplinary experts from our research team divided each full class video into “segments”, which became the unit of analysis in the 3D-LOP.

**Fig 1:**
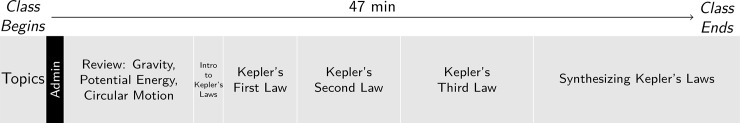
Example recording showing segments, the unit of analysis used in coding for the opportunity to engage students in 3DL. Segments from the recording of an example physics class session (47 minutes total) are identified by topic and depicted from class start to class end (left to right) by relative time duration (horizontal length represents the relative time of the segment with respect to the class session total).

A segment was defined as a temporally continuous portion of a class session consisting of a coherent discussion organized around the same topic(s). This approach was chosen, rather than using arbitrary units of time, because a 3D instructional segment often includes a sequence of instructional events that taken individually may not appear three-dimensional. Multiple types of classroom activities (described in the following Teaching Activities section) may be part of the same segment (e.g., lecture and clicker questions) so long as they were related by topic. Often, instructor cues were used to help identify segments (e.g., using phrases such as “reviewing from the previous class session” or “moving on to a new topic”), resulting in segments that lasted between approximately five and thirty minutes in length. In a typical 50-minute class the number of segments ranged from two to six. Fig 1 shows the segments from an example physics class session with segments depicted from class start to end by relative time duration.

In the rare case that a disciplinary expert tasked with segmenting a given video was uncertain, he or she sought input from another member of the research team. When coding for the three dimensions in a subsequent stage, coders had the opportunity to adjust the segments if necessary; such adjustment occurred for only one of the eighteen recordings included in this study (1 segment out of the original 71 was broken up; the single segment was into 4 smaller segments). An expanded discussion of the process for identifying segments is provided in the Supporting Information: 3D-LOP.

#### Coding segments for the three dimensions

Once the guidelines for dividing each recording into segments had been determined, we developed the coding criteria for each dimension. The criteria were developed through an iterative process consisting of both disciplinary and interdisciplinary discussions regarding the coding of 39 video recordings from biology, chemistry, and physics class sessions. This extended process involved the whole research team coding common recordings and disciplinary subgroups coding additional recordings to ensure calibration within and across disciplines throughout the development of the criteria as discussed below in the section on validity and reliability.

**Fig 2:**
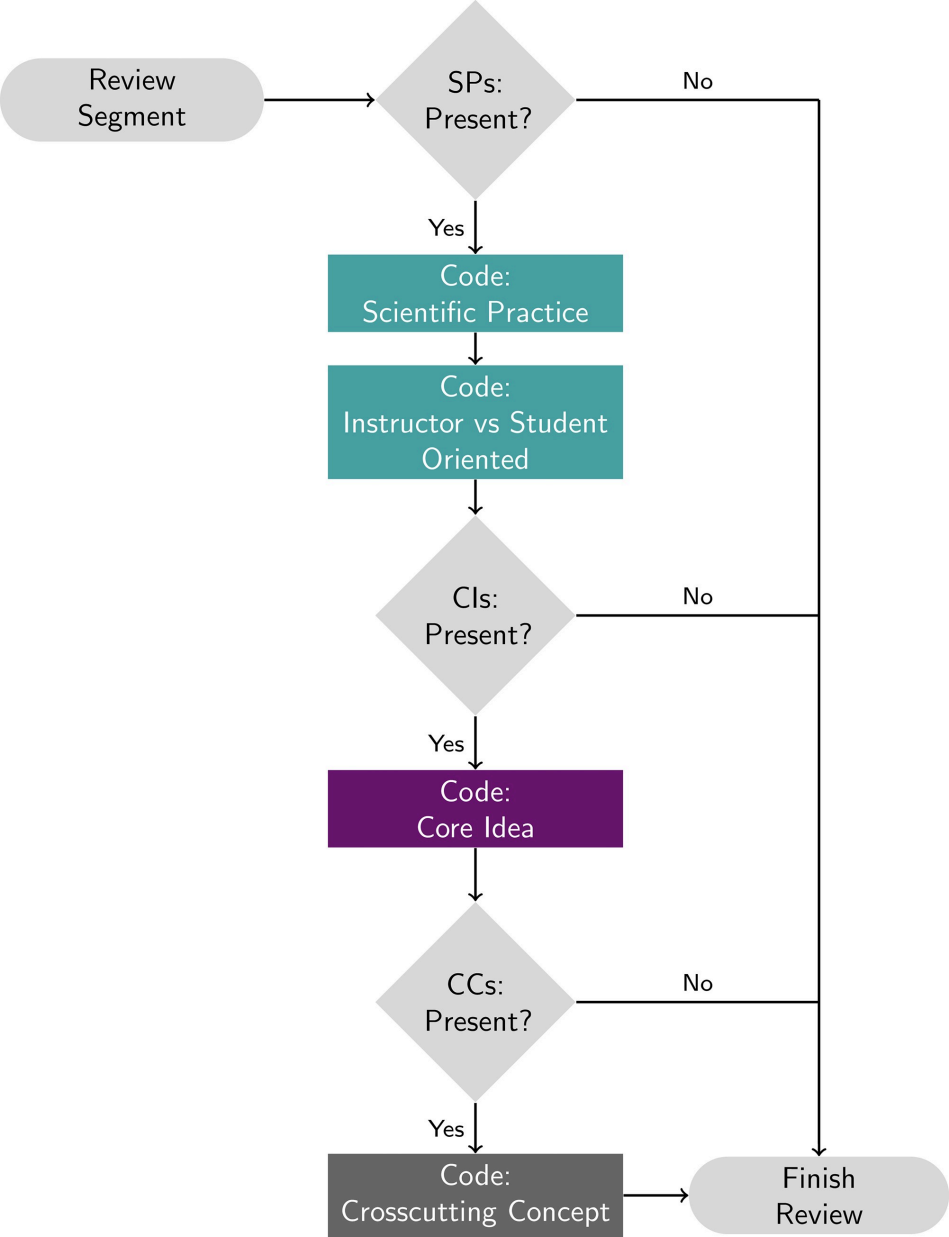
Summary of the three dimensions coding workflow for a single segment.

It quickly became apparent that coding video data for three-dimensionality is more complex than coding assessment items. To provide structure and support for the coders, an algorithmic workflow for coding video segments with the three dimensions was used as shown in Fig 2. Although we are ultimately interested in determining whether the instructional segment incorporates all three dimensions, we prioritized coding for the presence of a scientific practice because we determined that it was not feasible to reliably identify the presence of a core idea in the absence of a practice. That is, unless knowledge is used to do something (e.g., explain, model, analyze and interpret data), it is difficult to determine if a topic or phenomenon is *linked* to a core idea given that core ideas are concepts that are central to the discipline and that individual topics should be connected to these ideas to support the development of a more expert-like framework. The linkages between topics and core ideas can become more apparent when scientific practices are present. Arguably, if experts cannot agree on whether a core idea is present in the absence of a scientific practice, it is unlikely that a student could identify that a topic or phenomenon is being tied to a core idea.

The vision of three-dimensional instruction is to promote the process of developing and using connected knowledge. Therefore, when characterizing a class session using the 3D-LOP, the coder first considers if the instruction within a single segment reflects engagement with a scientific practice (Fig 2). The presence of a practice is relatively straightforward to identify, particularly when adapting the criteria developed originally for the 3D-LAP (Table 3). For example, many class sessions focus on extensive calculations (often called problem solving), but if the calculation does not lead to some consequence or interpretation of the numerical answer, the activity of calculating is not coded as using mathematical thinking. Similarly, if students are asked to draw chemical structures, the activity is not coded as developing and using models unless those structures are used to make a prediction about or explain an observation or phenomenon with the reasoning that explicitly connects the model to the prediction or explanation.

**Table 3:** Coding criteria for two example scientific practices from the 3D-LOP.

Developing and Using Models	Using Mathematics and Computational Thinking
Students are given or asked to construct a mathematical, graphical, computational, symbolic, or pictorial representation and use it to explain or predict an event, observation, or phenomenon. 1. Instruction presents an event, observation, or phenomenon for instructor/students to explain or make a prediction about. 2. Instruction presents a representation or asks instructor/students to construct a representation. 3. Instruction has instructor/students **explain or make a prediction** about the event, observation, or phenomenon. 4. Instruction has instructor/students provide the **reasoning that links the representation to their explanation or prediction.**	Students are asked to use mathematical reasoning or a calculation and interpret the results within the context of the given event, observation, or phenomenon. 1. Instruction presents an event, observation, or phenomenon. 2. Instruction has instructor/students perform a calculation or statistical test, generate a mathematical representation, or demonstrate a relationship between parameters. 3. Instruction has instructor/students **give a consequence or an interpretation** (not a restatement) in words, diagrams, symbols, or graphs of the mathematical results while demonstrating reasoning in the context of the given event, observation, or phenomenon.

The criteria for the practices that are often missing in instruction that is not three-dimensional are bolded.

If a practice is present, the coder also characterizes whether it is primarily the instructor or the student who is engaging in this practice (described in the following subsection). At this point, the coder determines if the instruction in the segment reflects a core idea and again, if yes, the coder goes on to the final dimension, the crosscutting concept (Fig 2). If at any point the segment does not reflect a dimension, that segment is not further coded. By moving the scientific practices to the forefront of the coding scheme, characterizing instruction with the 3D-LOP *prioritizes the use of knowledge* in science courses. As with the 3D-LAP, the 3D-LOP criteria for each dimension take somewhat different forms. Each scientific practice has two to four required criteria, each crosscutting concept has one to four criteria, and each core idea is described in detail. The full protocol is provided in the Supporting Information: 3D-LOP.

#### Instructor and student engagement in scientific practices

Reviewing many video recordings across disciplines and instructors revealed a range of approaches to incorporating scientific practices in instruction, influenced by factors such as the instructor, curriculum, and class size. Therefore, the 3D-LOP criteria are also designed to characterize who is “doing the work” of the scientific practice. This is accomplished by denoting whether engagement with the scientific practice is more instructor-oriented or student-oriented along with the scientific practice code for a given segment. This distinction is based on the instructor and student actions during a given segment, particularly noting who is expected to do what. Many of the practices have criteria that require some form of reasoning. For example, as described above, to code a segment with the practice of developing and using models, it would not be sufficient to have students draw a chemical structure (which is rules-based); the structure would also need to be used to predict and explain physical or chemical properties. For a student-oriented designation, the students must contribute a majority of the reasoning, and for the instructor-oriented designation, the instructor would provide the reason that the structure leads to certain physical or chemical properties. The complete criteria for coding instructor and student engagement in scientific practices can be found in the Supporting Information: 3D-LOP.

### Teaching activities

Each video was also coded for the sequence of activities occurring during the class meeting (i.e., how the class is being taught) to provide insight into how each class session was facilitated (Table 4). The coding criteria for these teaching activities [16] were developed through team discussions informed by published criteria from the COPUS, TDOP, and RTOP [23–25]. This teaching activity coding is independent of the segmenting by a disciplinary expert; that is, multiple types of teaching activities could occur during a single segment. For example, clicker questions, a task, and a short lecture might all be part of the segment defined by the disciplinary expert. The full criteria for the teaching activity coding can be found in the Supporting Information: 3D-LOP.

**Table 4:** Description of selected teaching activities coded with the 3D-LOP.

Teaching Activity	Description
Administration	The instructor informs the students about news items and general course business, for example, material related to how the course is run, homework deadlines, and exam information.
Clicker Question	The instructor provides a multiple-choice question that students respond to using a audience-polling system.
Lecture	The instructor presents information to the students relevant to the topic of study for that class session.
Task	The instructor asks the students to engage in an activity alone or with their classmates relevant to the topic of study for that class session.

### Reliability

Here, we outline the steps taken to establish validity and reliability for the 3D-LOP in the context of our instructional transformation efforts at MSU. As with all protocols of this nature, others should conduct their own validity and reliability measures for their datasets, as validity and reliability are constructs dependent upon both the tools and data [30]. Both the development and the inter-rater reliability were conducted using data from our dataset in order to establish these measures.

The 3D-LOP protocol was developed through extensive negotiation between all of the authors that included numerous rounds of immersion in the data. The discussions and decisions made in these iterative rounds were grounded in the Framework, our prior work developing the 3D-LAP, and practice (e.g., video recordings of classroom instruction). Each of these steps were taken to ensure face and content validity for the 3D-LOP protocol with our dataset. To conduct this work, the team coded about 20% of our data corpus (39 of 181 recordings), discussing results and resolving discrepancies as we developed the coding process.

The reliability of the 3D-LOP with our dataset was assessed by coding six video recordings per discipline (18 recordings total, ~10% of the dataset); these 18 recordings were in addition to the 39 selected and used for the development of the 3D-LOP. Each discipline’s six recordings were selected to capture both traditional and transformed instruction. Here, traditional and transformed instruction were defined based on our prior work using the 3D-LAP; recordings were selected largely based on the fraction of exam points that were 3D. Each previously segmented video was coded for the three dimensions by two disciplinary experts from the research team, and each coder pair was unique for every video. The resultant coding was used to calculate inter-rater reliability based on percent agreement. Coders were considered to be in agreement on the dimensions if their coding characterized a segment as 3D or not. While it is possible to compare coding for each dimension (present or absent as well as the specific practice, core idea, or crosscutting concept), we contend that ultimately it is the 3D nature of the instruction that is important due to the nature of our research interests and mode of reporting (fully 3D segments versus non-3D segments).

As with the 3D-LAP, due to the overlap between many of the practices and core ideas, coders did not necessarily need to agree on the *specific* scientific practice, core idea, or crosscutting concept when coding using the 3D-LOP criteria; rather, they needed to agree on the presence or absence of a given *dimension*. For example, an activity that asks students to draw molecular-level pictures of an ionic compound before and after dissolution, describe the forces present in each scenario, discuss the relative strengths of those forces, and explain the observed temperature change of a given dissolution process using the drawings and forces could reasonably be coded as either the scientific practice of *Developing and Using Models* or *Constructing Explanations and Engaging in Argument from Evidence* because the segment meets the criteria for each. Likewise, multiple core ideas (Electrostatic and Bonding Interactions; Energy: Atomic/Molecular and Macroscopic) and crosscutting concepts (Cause and Effect; Systems and System Models; Energy and Matter) could reasonably be coded.

The inter-rater reliability measured between coders, as well as the number of coders and segments, is reported in Table 5. If the agreement between two coders was below 70%, a third coder was added. This addition was necessary for three of the eighteen recordings (one in biology and two in chemistry). The addition of a third coder accounts for the differences between the totals in the third and fourth columns of Table 5; that is, the number of segments in the six recordings per discipline does not necessarily equal the total number of possible pairwise agreements. A lower limit of 75% for IRR was set [13]. However, the 67% instructor versus student agreement reported for the physics recordings was considered acceptable because of the small number of 3D segments on which to compare agreement; only three out of 24 segments across the six physics recordings reflected a scientific practice.

**Table 5:** Coding reliability.

Discipline	Number of Coders	Number of Segments	3D Agreement	I vs. S Agreement
Biology	4	26^a^	25 of 32 (78%)	10 of 11 (91%)
Chemistry	7	24^a^	31 of 38 (82%)	15 of 15 (100%)
Physics	6	24^a^	24 of 24 (100%)	2 of 3 (67%)
Overall	17	71^b^	80 of 94 (85%)	27 of 29 (93%)

Pairwise percent agreement used to determine inter-rater reliability in applying the 3D-LOP to characterize instruction in video recordings. The addition of a third coder for one in biology video and two chemistry videos accounts for the difference in segment totals in columns three and four (i.e., the number of segments in the six recordings per discipline do not necessarily equal the total number of possible pairwise agreements).

^a^Number of segments within the 6 recordings per discipline

^b^Number of segments within the 18 recordings overall

#### Determining validity: Can the 3D-LOP detect the potential to elicit 3DL instruction?

**Fig 3:**
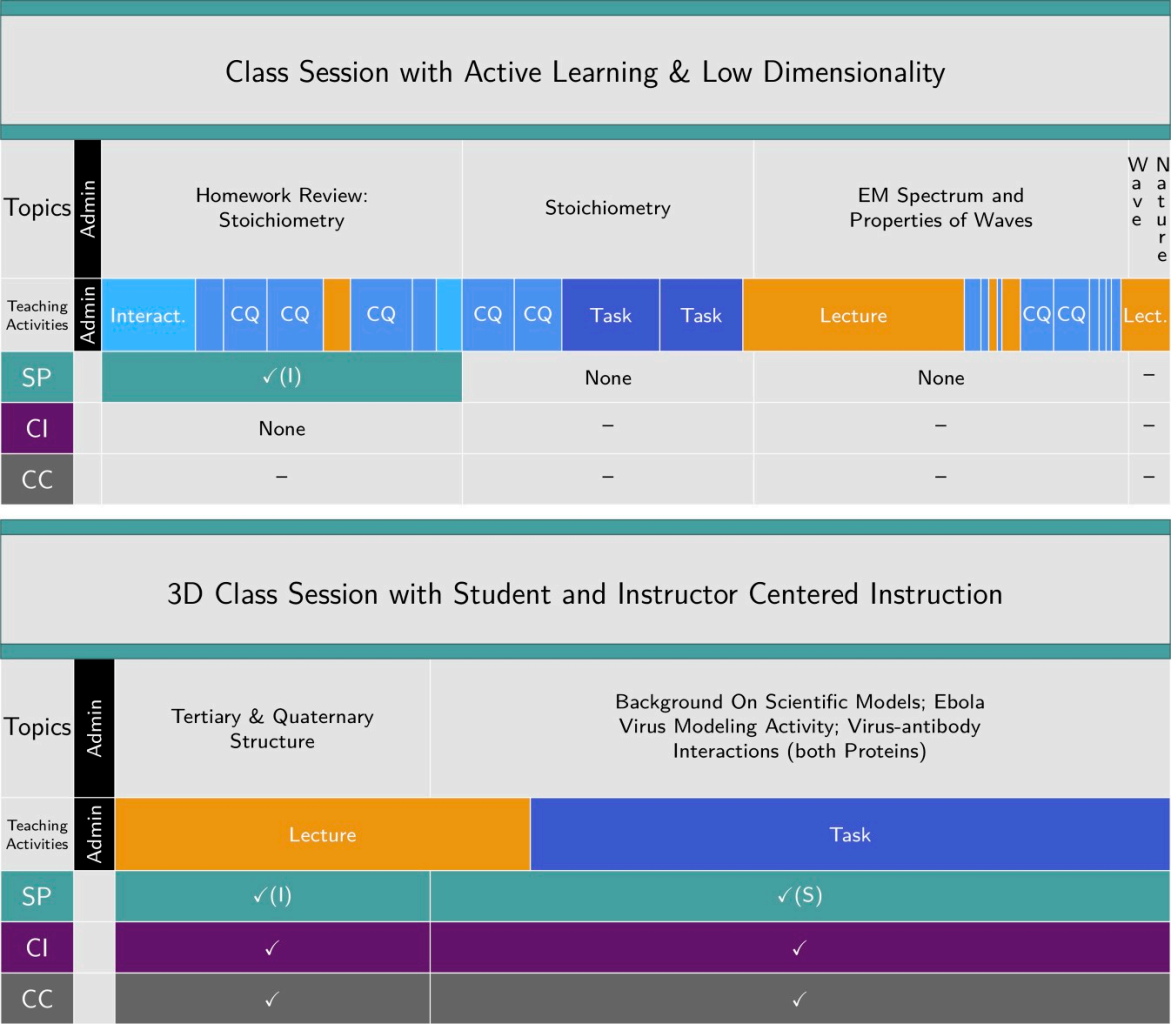
Complete timeline. Compiled timeline showing topics by segment, the coding for teaching activities (administration, lecture, tasks, and clicker questions (CQ)), and dimensions (scientific practices, core ideas, and crosscutting concepts). An “I” (“S”) for the scientific practice register denotes instructor- (student-) centered engagement with the practice. The top panel shows an example from a 79-minute chemistry class session with no 3D segments despite significant use of active learning techniques (Tasks, Clicker Questions, and Interactions; darker to lighter blue teaching activities). The bottom panel shows an example from an 80-minute biology class session with an instructor-centered 3D segment without active learning techniques employed, as well as a student-centered 3D segment.

Combining the teaching activity and dimension coding into a single timeline provides rich insight into the instruction during a given class session. Fig 3 depicts two compiled 3D-LOP timelines, one for a chemistry class session and one for a biology class session, revealing the topics addressed during instruction (as shown in Fig 1), how the class was facilitated (teaching activity coding), and engagement in the dimensions (dimension coding, Fig 2). The timelines can be used to characterize the extent to which the instructors and students are engaging with each of the three dimensions, as well as how this engagement is being facilitated. More examples of coded recordings from biology, chemistry, and physics class sessions are provided in the Supporting Information: Exemplars.

In addition to demonstrating measures of credibility for the 3D-LOP, our goal was to determine whether the protocol could distinguish between instruction that has potential for engaging students with the three dimensions outlined in the Framework and instruction that does not. The 18 recordings (six from each discipline) were de-identified and coders were not aware of any prior results from the 3D-LAP. Each video was coded by two individuals: both experts in the discipline of the class who were members of the research team. Independent coding was used to determine initial agreement between coders. Coders then met to achieve consensus on the coding for each video to use when reporting the final characterization of a class session. In the four cases where agreement between two coders was not reached, a third team member was consulted to assist in making the final decision.

**Fig 4:**
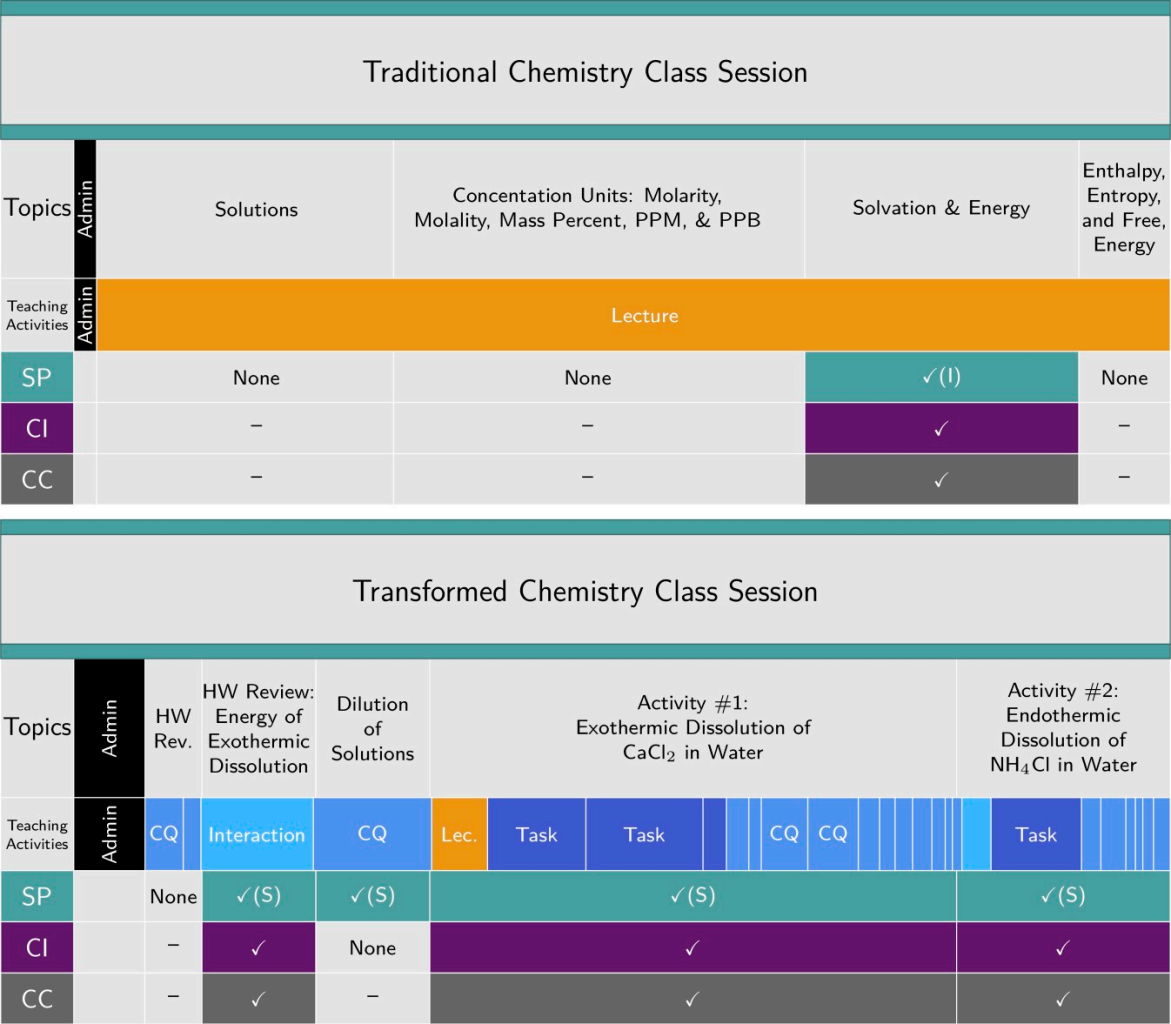
Comparing a traditional and a transformed class session. Comparison of a traditional chemistry class session (47 minutes) to a transformed chemistry class session (77 minutes), where the instructor and topics were the same but recorded two years apart (before and after course transformation). The timelines depict coding for teaching activities (administration, lecture, tasks, clicker questions (CQ)) and dimensions (scientific practices, including instructor-centered (I) vs. student-centered (S) engagement, core ideas, and crosscutting concepts).

Fig 4 compares the timelines for two chemistry class sessions, one associated with a traditional curriculum and one from the course that follows a transformed curriculum [31]. In these recordings, the same instructor taught the same topic two years apart, before and after course transformation. The percent of 3D exam points was assessed using the 3D-LAP, revealing an increase from 0% to 47% in the semesters when these recordings were generated. The 3D-LOP dimension coding reveals that the transformed class session had ample potential for engagement with the dimensions, whereas the traditional class session had little. The teaching activity coding shows that the instructor changed pedagogical strategies when implementing 3D instruction compared to the traditional instruction. That is, in the transformed class session in Fig 4, there were many more opportunities for interaction via student-driven tasks and clicker questions, whereas in the traditional session the instruction centered around lecture. During the part of the traditional lecture where there was the potential to engage in 3DL, it was, in fact, the instructor who was doing the “work”.

**Fig 5:**
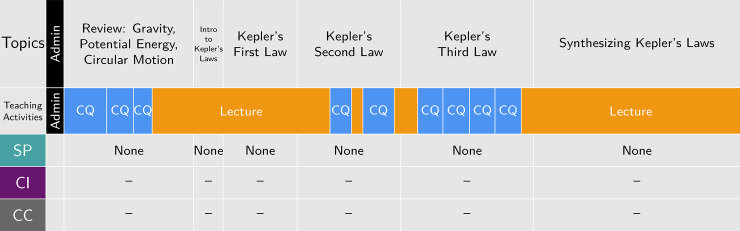
Example active class session that does not reflect 3DL. Example physics class session (47 minutes) where the 3D-LOP coding characterizes instruction with teaching activities that are more student-oriented than in many traditional instructional settings but no engagement with scientific practices is evident. The timeline depicts coding for teaching activities (administration, lecture, and clicker questions (CQ)) and dimensions (scientific practices, core ideas, and crosscutting concepts).

Increased student engagement (as shown through teaching activity coding) did not always correlate to 3D instruction (as shown through dimension coding). For example, Fig 5 shows the timeline from an example physics class session. Clicker questions were used throughout, but the instruction did not demonstrate potential for engagement with scientific practices. In this class session, the students were engaged in numerical problem-solving exercises. However, because the answers were neither used to explain a phenomenon nor re-expressed in another form as required to satisfy the criteria for the practice of *Using Mathematics and Computational Thinking*, this ostensibly active class did not reflect 3DL. Additional evidence supporting the ability of the 3D-LOP to distinguish between instruction that has the potential for engagement with the three dimensions is provided in Supporting Information: Exemplars. In future work, our team intends to use the 3D-LOP to investigate change over time in instruction to evaluate the extent of transformation in biology, chemistry, and physics courses. This will complement previous efforts to evaluate the transformation of assessments with respect to their potential to engage students in 3DL over time in these same courses, and to examine the relationship between changes in assessments and instruction [16].

## Discussion

The goals of this paper are to introduce the 3D-LOP and provide evidence that it can be used to characterize instruction that has the potential to engage students in 3DL within the context of introductory biology, chemistry, and physics courses. We have demonstrated that we are able to reliably code for the presence of 3DL, and that we can also differentiate between instruction that has the potential to promote three-dimensional learning from instruction that does not as shown in Fig 4. Our goals for the use of this protocol are two-fold: we plan to use it to evaluate outcomes of instructional transformation projects, and we also propose that it can be used to support practitioners in developing curricular materials and selecting instructional strategies to promote engagement in three-dimensional instruction. For example, it could be used as a peer observation tool and to identify both 3DL exemplars and missed opportunities for inclusion of 3DL to support professional development activities.

The 3D-LOP serves to advance the teaching and learning of science in that it provides the means to characterize not only *how* a class is taught, but also the nature of *what* is being taught by identifying opportunities for students to demonstrate their understanding of core ideas through engagement with scientific practices. The 3D-LOP goes beyond identifying the various teaching activities occurring during a class, such as clicker questions and tasks that often fall under the banner of “active learning”. In our context, we plan to use the 3D-LOP to evaluate course transformation efforts at Michigan State University, Florida International University, Grand Valley State University, and Kansas State University by characterizing whether changes in instruction with respect to 3DL have occurred over time. This characterization will provide a comprehensive picture of transformation efforts, complementing a prior change over time analysis that used the 3D-LAP to characterize exams [16].

We do not advocate that all instruction should be 3D, just as we do not suggest that all assessment items should be 3D [13,15,16,20]. However, if 3DL is deemed important and central to course transformation, all aspects of a course, including instruction, should align. Students must have adequate exposure to and opportunities to engage with 3DL in formative settings as part of classroom instruction and activities outside of the classroom (e.g., through homework) to prepare for summative 3D assessments. While the ultimate goal is student engagement in 3DL, certainly in large enrollment classes it may be necessary for instructors to model engagement with the practices, as well as provide opportunities for students to do so. Indeed, if learning objectives and course assessments hold student engagement and proficiency with the three dimensions as important, instruction must provide students the guidance and support to be successful.

It should be noted that this characterization of transformation is not linked to individual faculty data, and we do not recommend using the 3D-LAP or the 3D-LOP for evaluation of individual faculty. However, the 3D-LOP can also be used as a professional development tool to support faculty in developing instructional materials and selecting appropriate pedagogies that promote engagement with the three dimensions. Existing materials can be modified using the 3D-LOP criteria to guide decisions, providing faculty with a roadmap to 3D learning and lowering the barrier to transformation. The 3D-LAP and 3D-LOP are used by our research team in faculty professional development workshops on creating new and modifying existing course materials to promote adoption of 3DL, while providing support from these tools and expert users.

The development of the 3D-LOP and implementation of 3DL in college science courses, opens particular avenues for future inquiry. For example, studies about how students engage with and respond to 3DL can better guide the design of 3D instructional materials. It will be important to gather data on the impact of 3D instruction on student expectations and outcomes such as retention in STEM majors, time to graduation, and success of diverse student groups. Finally, we need to understand the barriers and levers to faculty adoption of 3D instruction.

As we move forward with these transformations and gain more understanding about how and when 3DL can be implemented, we hope to extend the 3DL framework to upper-level courses and use it to align the curriculum. Doing so will allow students to see how core ideas underlie their increasingly sophisticated and deeper understanding of the discipline, within the context of engaging with scientific practices. Further, it will allow students to reocgnize how crosscutting concepts allow us to focus on aspects of phenomena both within and across disciplines.

### Beyond 3DL: Knowledge-in-use

While our transformation efforts are focused on 3DL, we understand that others may be using different (and often complementary) approaches. While active learning has been used as an overarching term for instructional approaches that engage students, there is no guarantee that such approaches are actually promoting the use of knowledge. Any strategy that goes beyond active learning to emphasize knowledge-in-use could adapt the 3D-LOP to characterize instruction by using the approach described here to identify scientific practices. If instruction is centered around scientific practices, it will necessarily require students to use their knowledge. Therefore, the part of the of the 3D-LOP that focuses on scientific practices could be used to supplement more commonly used protocols, such as the COPUS [23], while providing the researcher with the ability to detect whether students are using their knowledge in a meaningful, more expert way or if they are engaging with tasks that require only memorization, pattern recognition, or rote problem-solving skills.

### Limitations

We reiterate that the 3D-LOP characterizes the potential for instruction to promote engagement in three-dimensional learning. It does not measure actual engagement (e.g., what the students are actually doing, such as having off-topic conversations or discussing presented materials in a meaningful way). Additionally, the 3D-LOP characterization does not infer what faculty intended for their instruction. Application of the 3D-LOP relies on observation of what the instructor says and makes visible to the entire class, such as displaying slides, writing on a blackboard, whiteboard, or document camera. A worksheet for an in-class activity distributed to students would not be captured unless the instructor discusses the work that students are engaging in. These measures must come from other data sources, such as instructor interviews or materials provided to students during class. To add to our evaluation of course transformation, interviews of instructors by our research team are ongoing.

Because entire segments in each recording are characterized as 3D (or not), there may be the potential to overstate the amount of 3D instruction within a class session if there is only a small portion of a segment that reflects 3D engagement. To date, instances in which this occurred in our coding have been rare, but we acknowledge this may be an issue. When we have noticed this, we revisited the segmenting criteria to see if a reasonable argument can be made for re-segmenting the video to minimize the issue. However, segments were ultimately chosen as the unit of analysis because coding according to short, regular units of time frequently did not provide enough time for all of the necessary criteria for the dimensions to be met in a single, short time block. Segmenting recordings into these natural divisions allowed for sufficient time to satisfy all of the criteria for dimensions, and thus accurately reflect 3D engagement.

As a tool for coding 3D instruction, the 3D-LOP does not explicitly identify instruction where some, but not all, of the criteria for a scientific practice are present. However, by recording in the detailed coding which criteria for a practice are satisfied and which are not, one could provide a more detailed guide to adapting instruction to include a scientific practice. Finally, as a consequence of our choice to prioritize the scientific practices, situations where instruction is organized around a core idea or a crosscutting concept is addressed may not be detected.

The 3D-LOP was developed for use in evaluating instruction from gateway college-level biology, chemistry, and physics lecture courses at MSU. Our team and others are evaluating its fitness for application to courses at other levels and in other instructional environments, as well as in other science disciplines. We plan to extend our investigations of 3DL to upper-level undergraduate courses and are additionally extending the use of the 3D-LAP and 3D-LOP to evaluate exams and instruction at other academic institutions.

## Conclusion

While there is substantial work focusing on how to measure whether students are actively engaging with their learning, there has been less focus on *what* students are learning and *how* they are using that knowledge. In this report, we present an approach that can either replace or supplement other classroom observation protocols by going beyond what students are doing in the classroom to consider the content and the use of knowledge. We have shown that the 3D-LOP can identify transformed classes where 3DL is in use and differentiate between classes where active learning is in place but where students are not engaging in scientific practices. The 3D-LOP can be used to both monitor change over time, that is, to identify whether 3DL transformations are propagating over time, and to support faculty development in the use of 3DL or knowledge in use.

## Supporting information

S1 Fig Example of the segments in a biology class session video. This figure illustrates the results of segmenting an instructional video.
(TIFF)

S2 Fig Example of the dimension coding for each segment in a biology class session video. This figure shows that each segment is coded using the dimension criteria.
(TIFF)

S3 Fig Example of the teaching activity coding in a biology class session video. This figure illustrates the teaching activity coding, which is independent of the segments for that video.
(TIFF)

S4 Fig Compiled 3D-LOP timeline of segmenting with coding for teaching activities and dimensions in a biology class session video. This figure brings together the various aspects of the analysis to depict the compiled 3D-LOP timeline.
(TIFF)

S5 Fig Flowchart to guide the coding of a segment using the 3D-LOP dimension criteria, where if the answer to the question in each arrow is “no”, stop coding the segment. This figure depicts the process of coding using the 3D-LOP dimension criteria.
(TIFF)

S6 Fig Traditional biology example 1. Introductory-Level Cell and Molecular Biology Class Session.
(TIFF)

S7 Fig Traditional biology example 2. Introductory-Level Cell and Molecular Biology Class Session.
(TIFF)

S8 Fig Traditional biology example 3. Introductory-Level Organismal and Population Biology Class Session.
(TIFF)

S9 Fig Transformed biology example 1. Introductory-Level Cell and Molecular Biology Class Session.
(TIFF)

S10 Fig Transformed biology example 2. Introductory-Level Cell and Molecular Biology Class Session.
(TIFF)

S11 Fig Transformed biology example 3. Introductory-Level Organismal and Population Biology Class Session.
(TIFF)

S12 Fig Traditional chemistry example 1. Introductory-Level General Chemistry II.
(TIFF)

S13 Fig Traditional chemistry example 2. Introductory-Level General Chemistry I.
(TIFF)

S14 Fig Traditional chemistry example 3. Introductory-Level General Chemistry I for Majors.
(TIFF)

S15 Fig Transformed chemistry example 1. Introductory-Level General Chemistry II.
(TIFF)

S16 Fig Transformed chemistry example 2. Introductory-Level General Chemistry II.
(TIFF)

S17 Fig Transformed chemistry example 3. Introductory-Level General Chemistry I.
(TIFF)

S18 Fig Traditional physics example 1. Introductory-Level Calculus-Based General Physics II.
(TIFF)

S19 Fig Traditional physics example 2. Introductory-Level Calculus-Based General Physics II.
(TIFF)

S20 Fig Traditional physics example 3. Introductory-Level Calculus-Based General Physics I.
(TIFF)

S21 Fig Transformed physics example 1. Introductory-Level Calculus-Based General Physics I.
(TIFF)

S22 Fig Transformed physics example 2. Introductory-Level Algebra-Based General Physics II.
(TIFF)

S23 Fig Transformed physics example 3. Introductory-Level Calculus-Based General Physics I.
(TIFF)

S1 File 3D-LOP protocol. This document includes an expanded description of the 3D-LOP protocol.
(DOCX)

S2 File Exemplars. This document contains timelines of class sessions from each discipline using video recordings captured before and after course transformations. Each class session timeline shows the segments, characterization of teaching activities, and coding from the 3D-LOP.
(DOCX)

S3 File Data. This document contains the individual coding described in this article.
(XLSX)
