# Supplementary figures and images for: Characterizing college science instruction: The Three-Dimensional Learning Observation Protocol
Source: PLoS One. 2020 Jun 16;15(6):e0234640. doi: 10.1371/journal.pone.0234640 (PMC7297354; doi:10.1371/journal.pone.0234640)

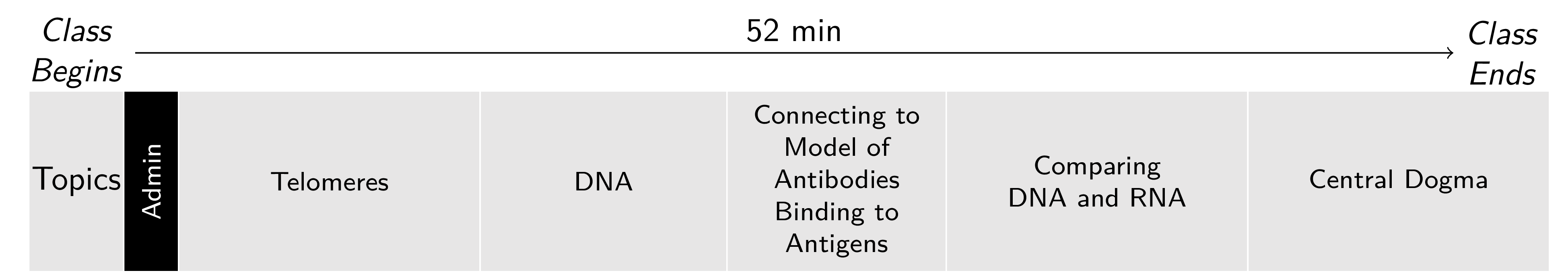

Supplement: S1 Fig — This figure illustrates the results of segmenting an instructional video. (TIFF) [file pone.0234640.s001.tiff]

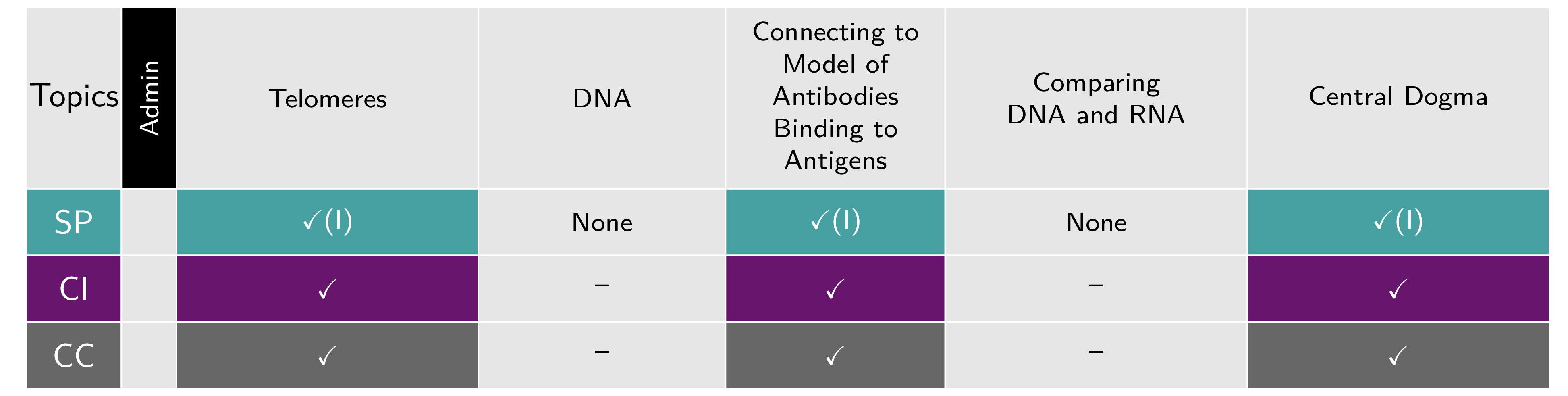

Supplement: S2 Fig — This figure shows that each segment is coded using the dimension criteria. (TIFF) [file pone.0234640.s002.tiff]

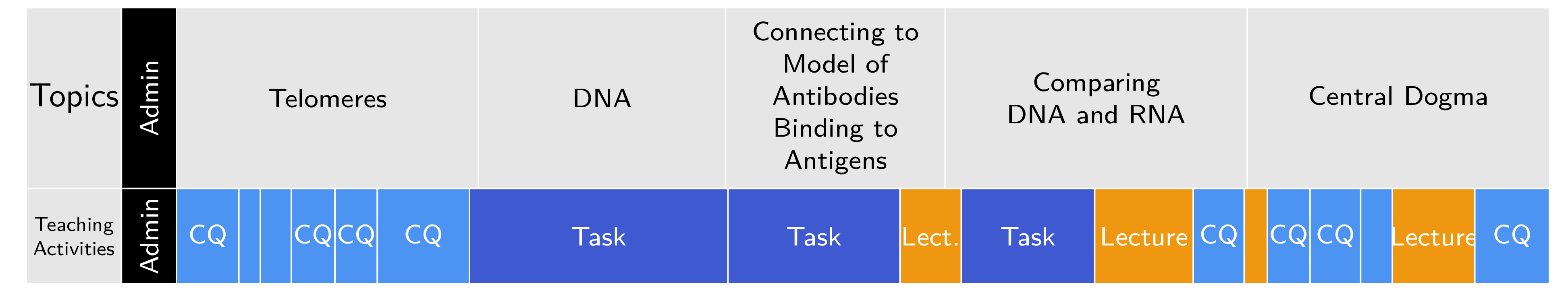

Supplement: S3 Fig — This figure illustrates the teaching activity coding, which is independent of the segments for that video. (TIFF) [file pone.0234640.s003.tiff]

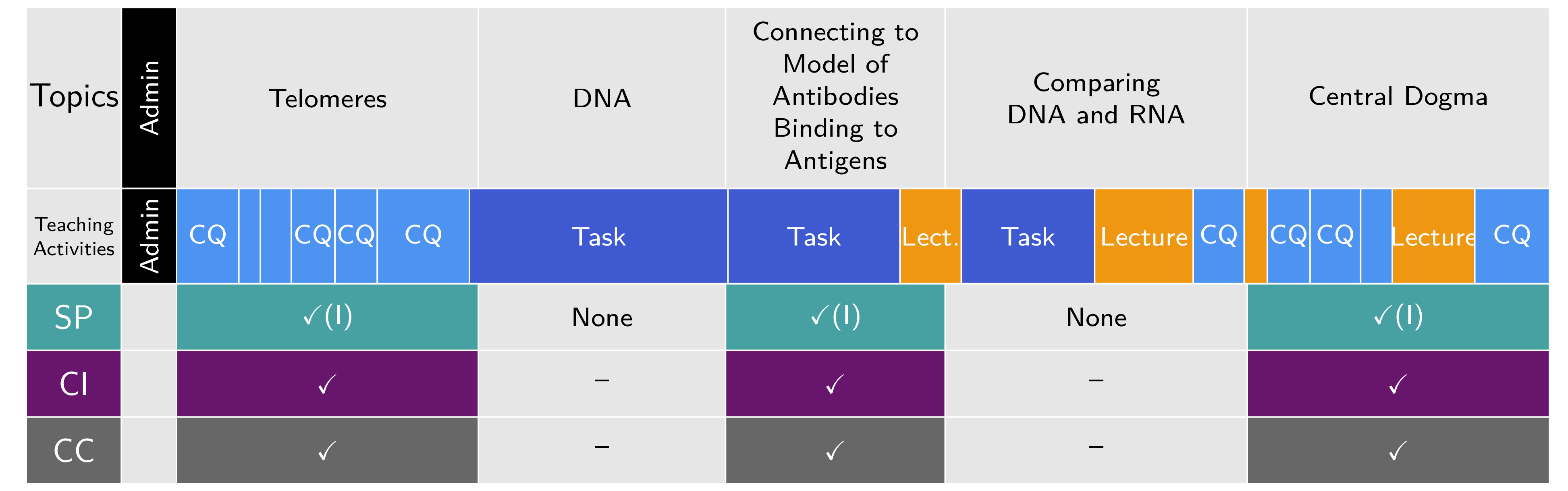

Supplement: S4 Fig — This figure brings together the various aspects of the analysis to depict the compiled 3D-LOP timeline. (TIFF) [file pone.0234640.s004.tiff]

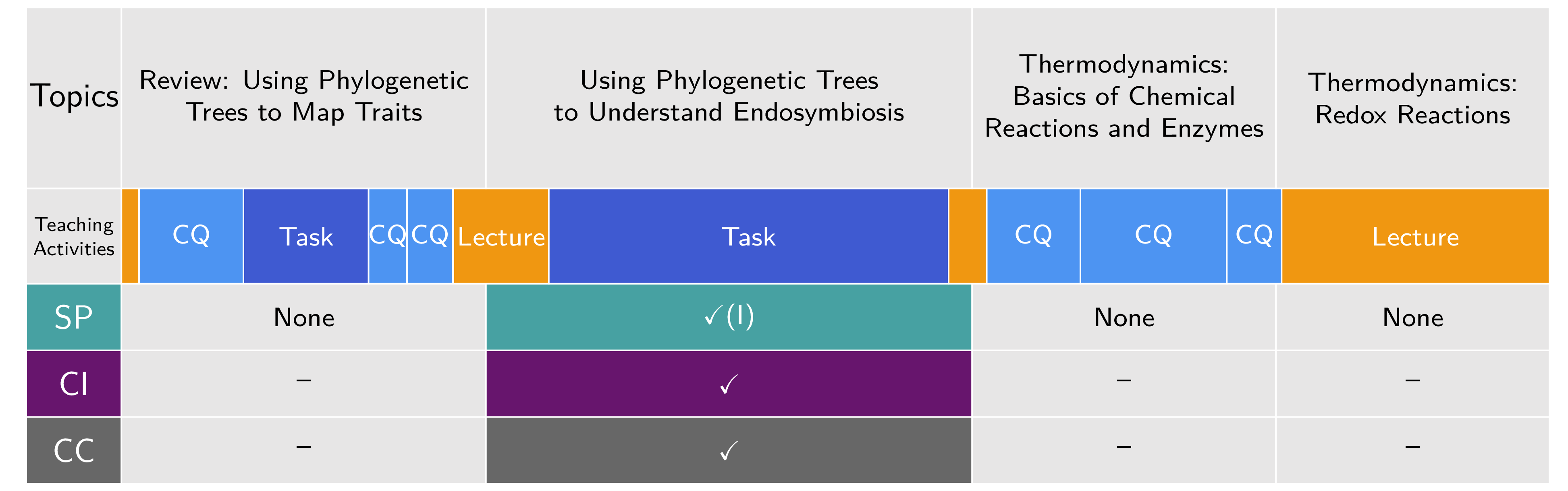

Supplement: S6 Fig — Introductory-Level Cell and Molecular Biology Class Session. (TIFF) [file pone.0234640.s006.tiff]

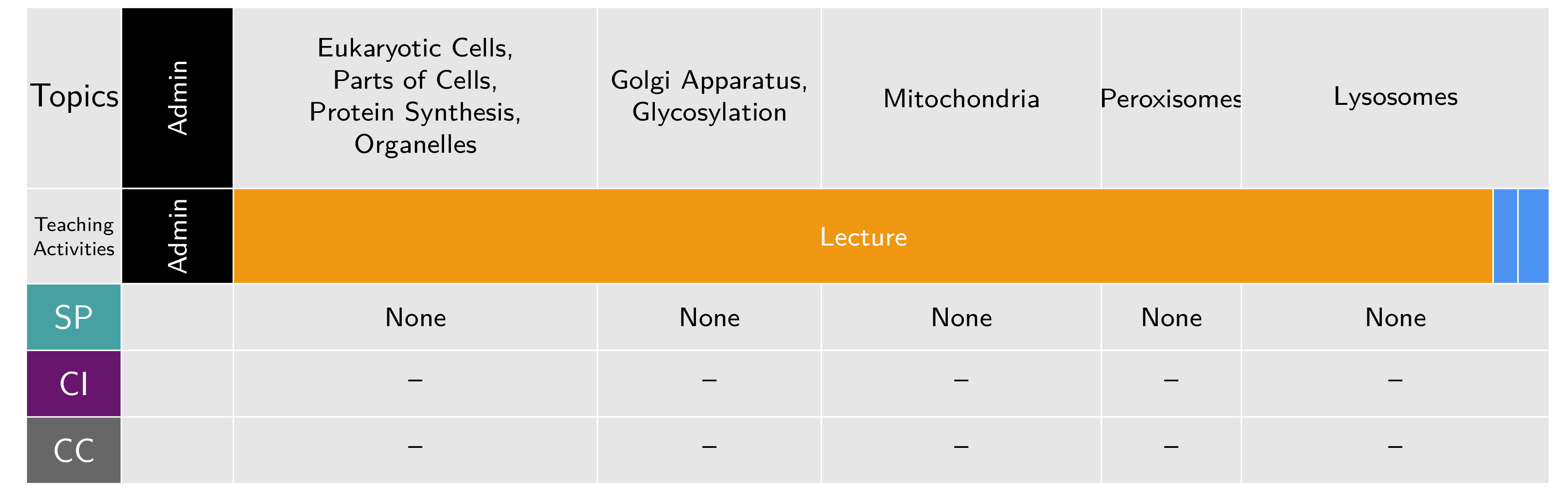

Supplement: S7 Fig — Introductory-Level Cell and Molecular Biology Class Session. (TIFF) [file pone.0234640.s007.tiff]

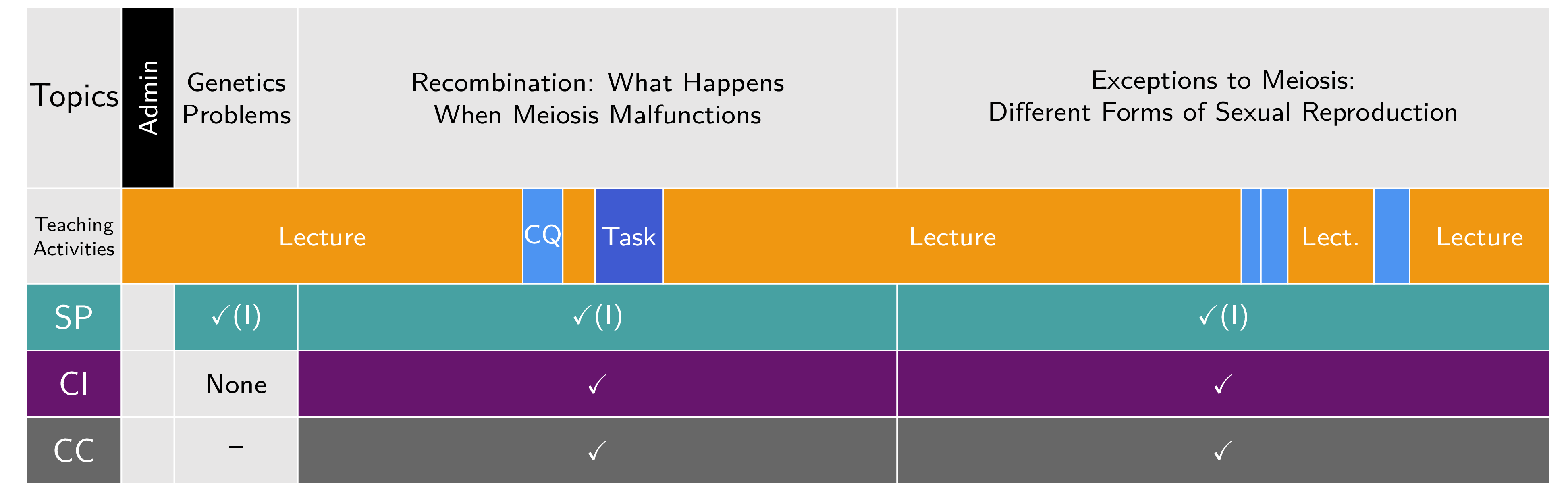

Supplement: S8 Fig — Introductory-Level Organismal and Population Biology Class Session. (TIFF) [file pone.0234640.s008.tiff]

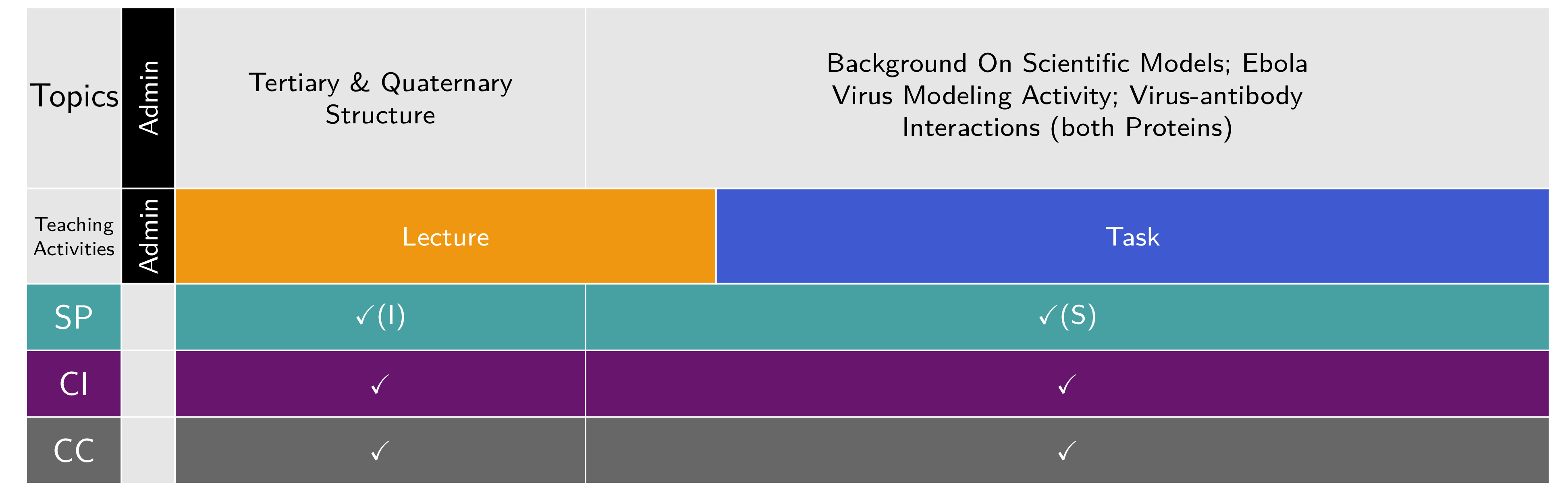

Supplement: S9 Fig — Introductory-Level Cell and Molecular Biology Class Session. (TIFF) [file pone.0234640.s009.tiff]

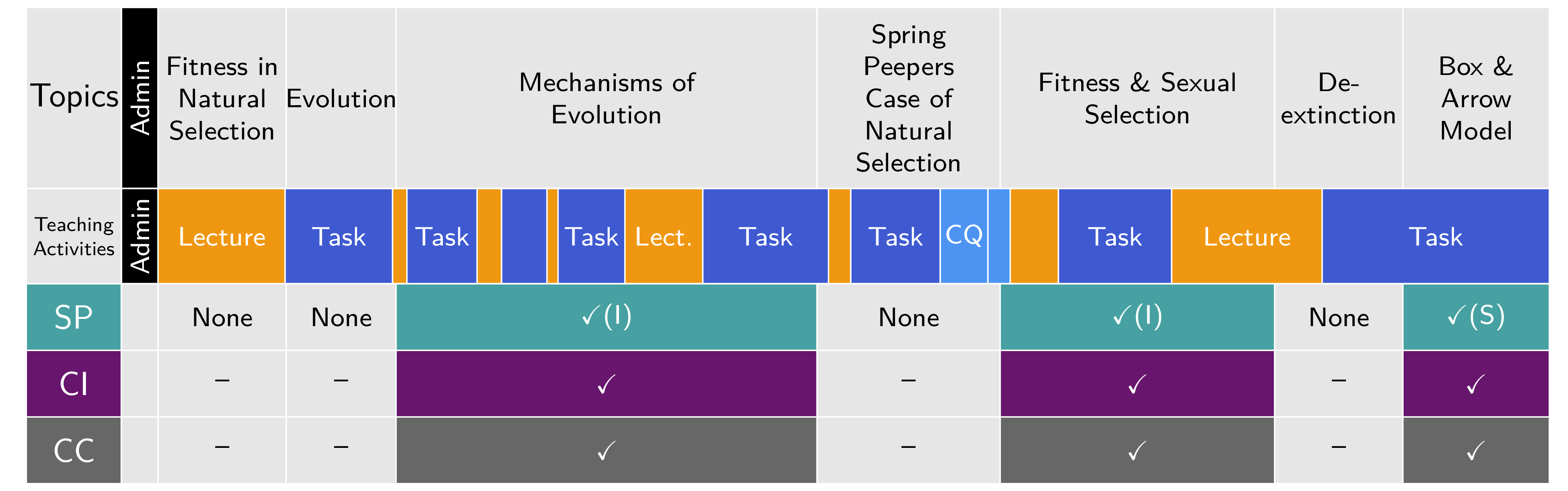

Supplement: S11 Fig — Introductory-Level Organismal and Population Biology Class Session. (TIFF) [file pone.0234640.s011.tiff]

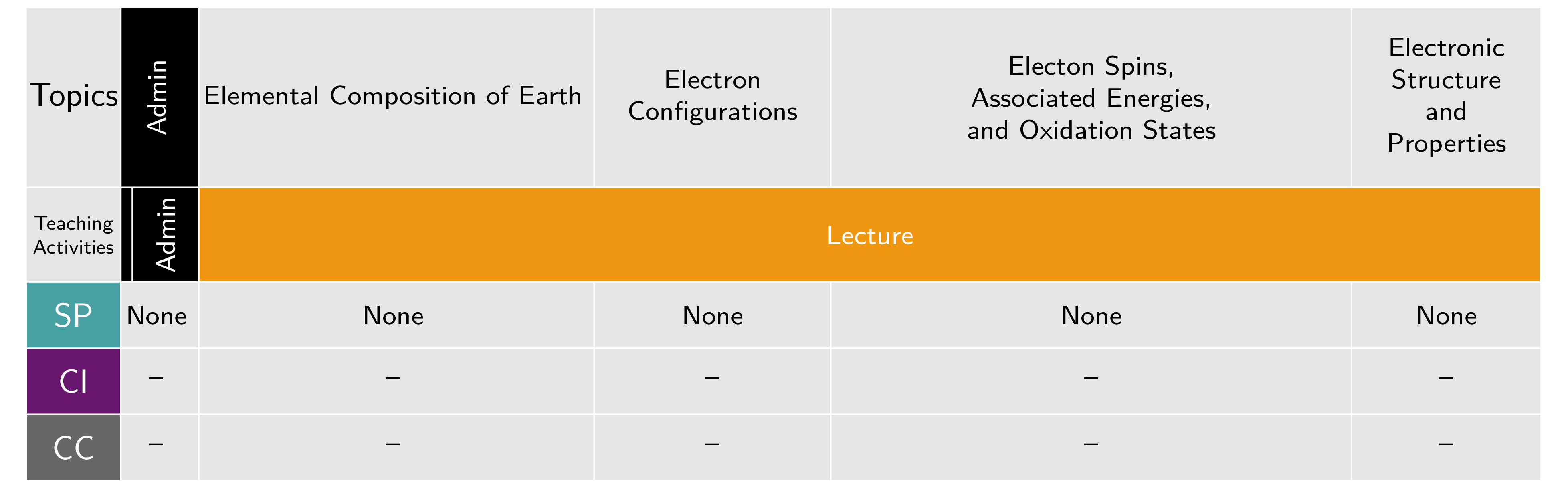

Supplement: S12 Fig — Introductory-Level General Chemistry II. (TIFF) [file pone.0234640.s012.tiff]

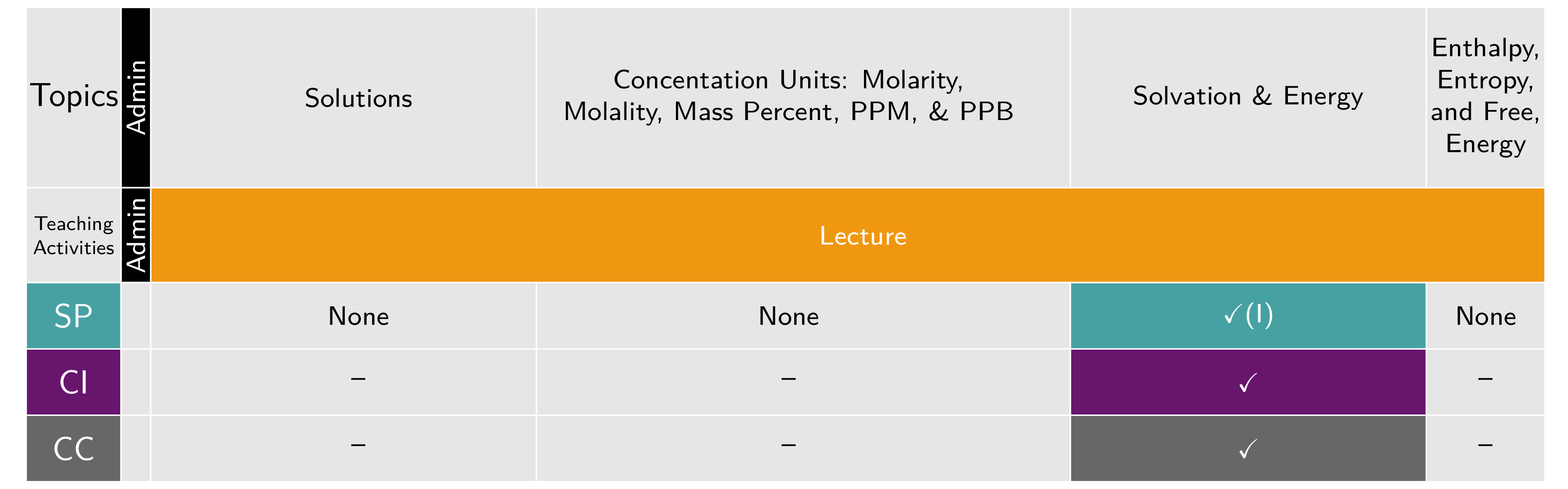

Supplement: S13 Fig — Introductory-Level General Chemistry I. (TIFF) [file pone.0234640.s013.tiff]

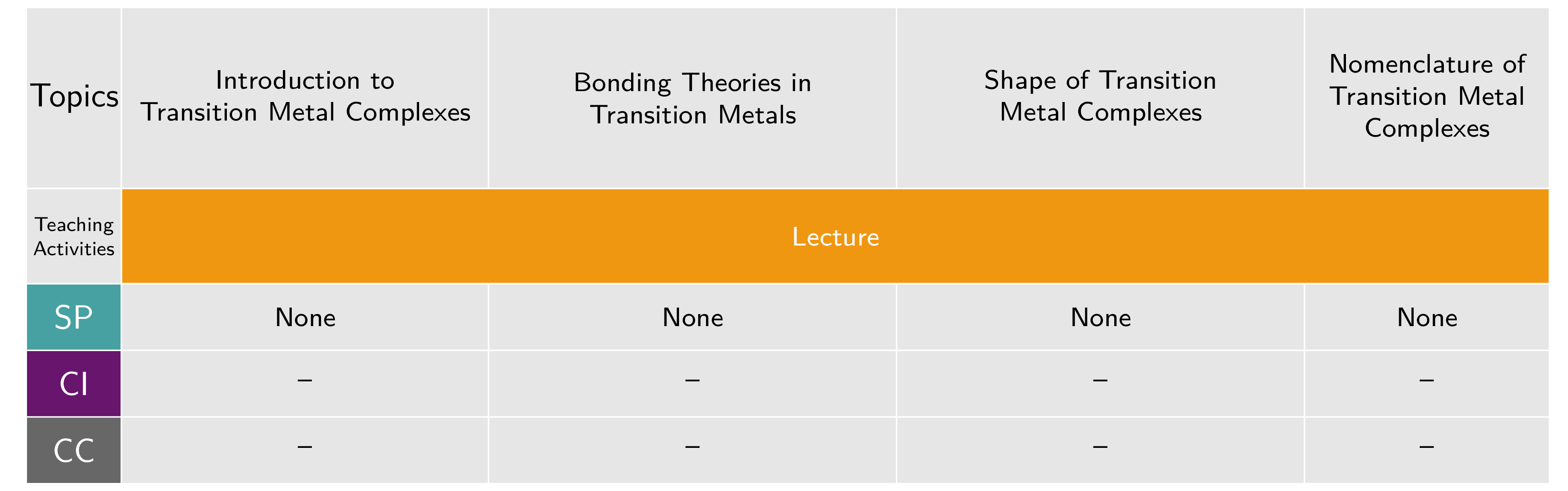

Supplement: S14 Fig — Introductory-Level General Chemistry I for Majors. (TIFF) [file pone.0234640.s014.tiff]

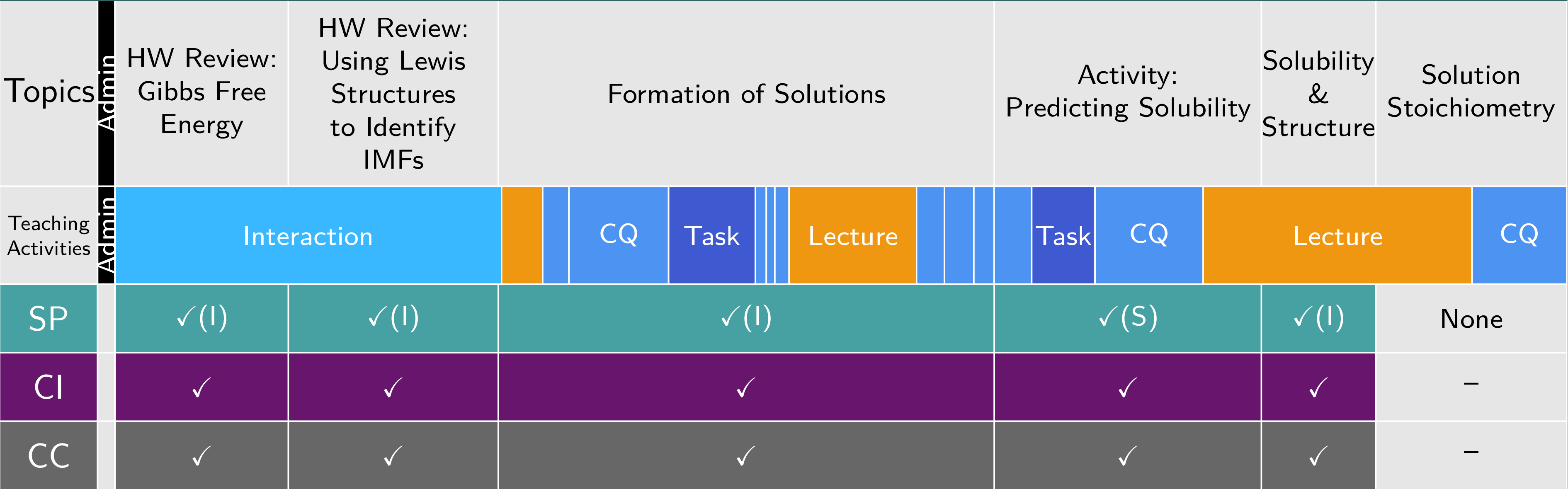

Supplement: S15 Fig — Introductory-Level General Chemistry II. (TIFF) [file pone.0234640.s015.tiff]

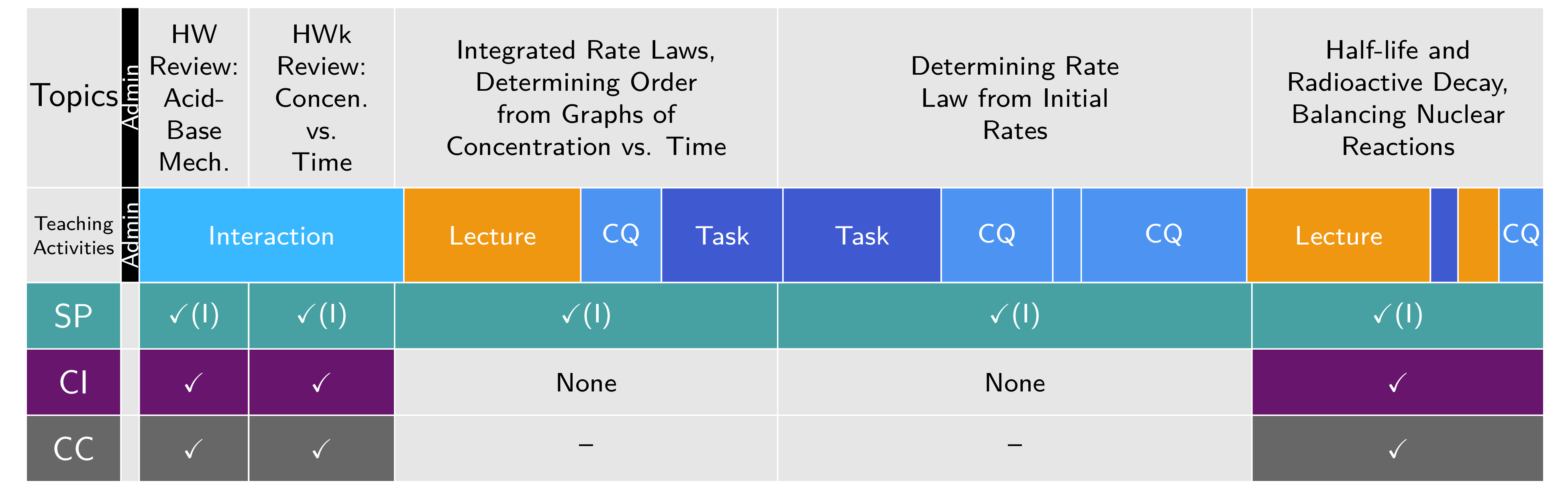

Supplement: S16 Fig — Introductory-Level General Chemistry II. (TIFF) [file pone.0234640.s016.tiff]

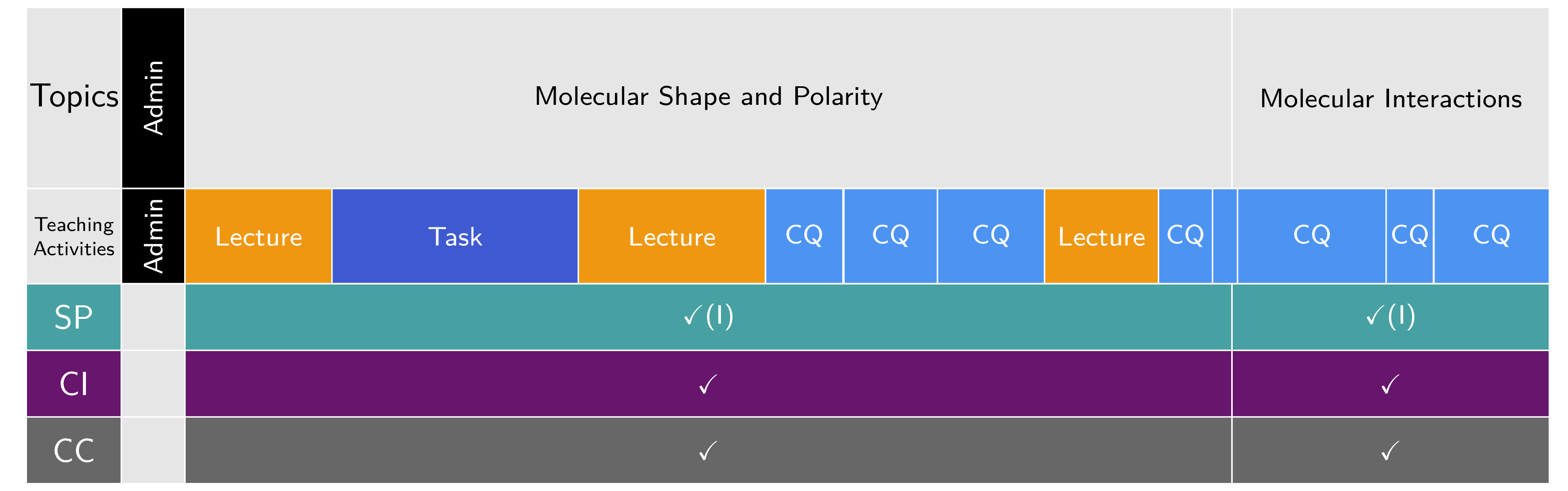

Supplement: S17 Fig — Introductory-Level General Chemistry I. (TIFF) [file pone.0234640.s017.tiff]

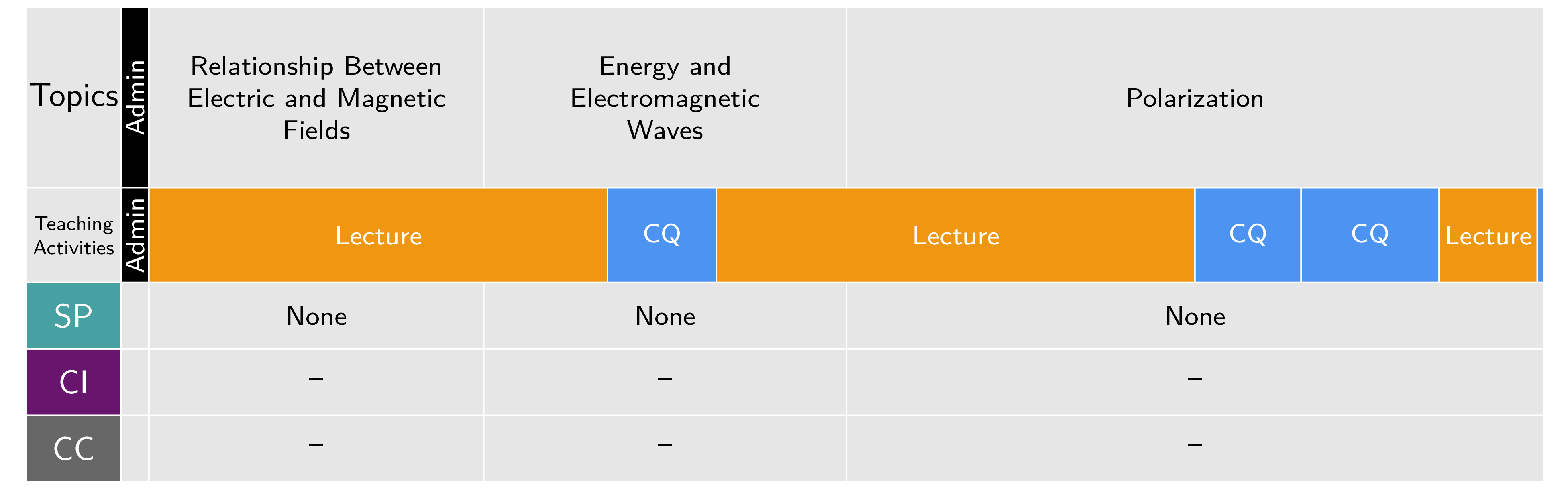

Supplement: S18 Fig — Introductory-Level Calculus-Based General Physics II. (TIFF) [file pone.0234640.s018.tiff]

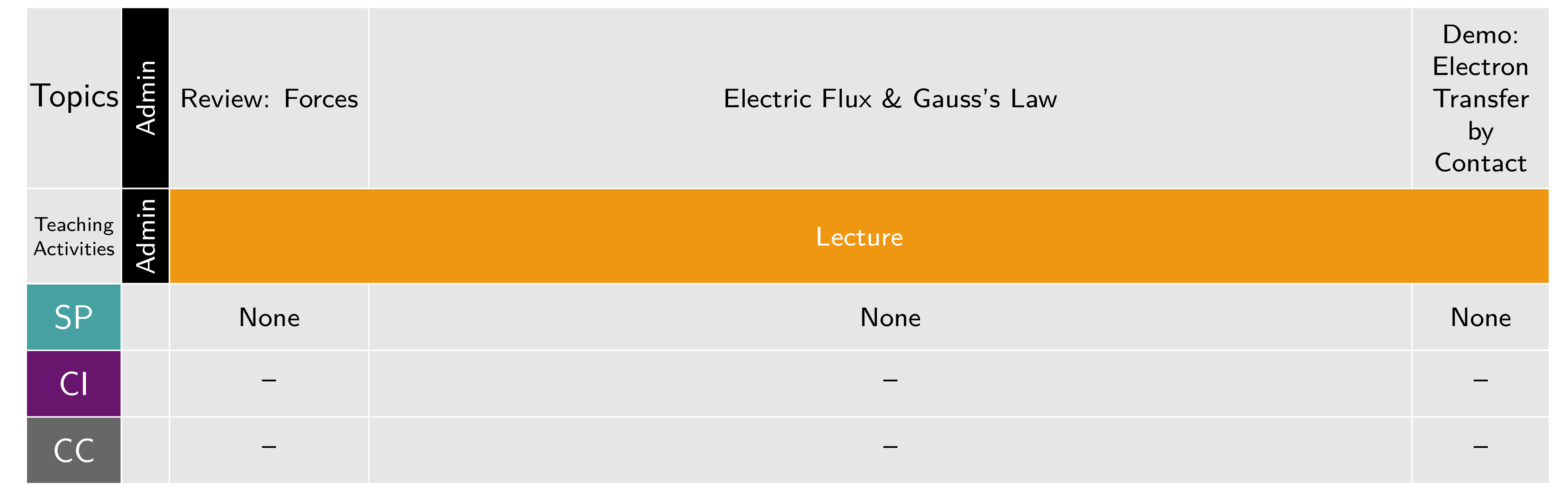

Supplement: S19 Fig — Introductory-Level Calculus-Based General Physics II. (TIFF) [file pone.0234640.s019.tiff]

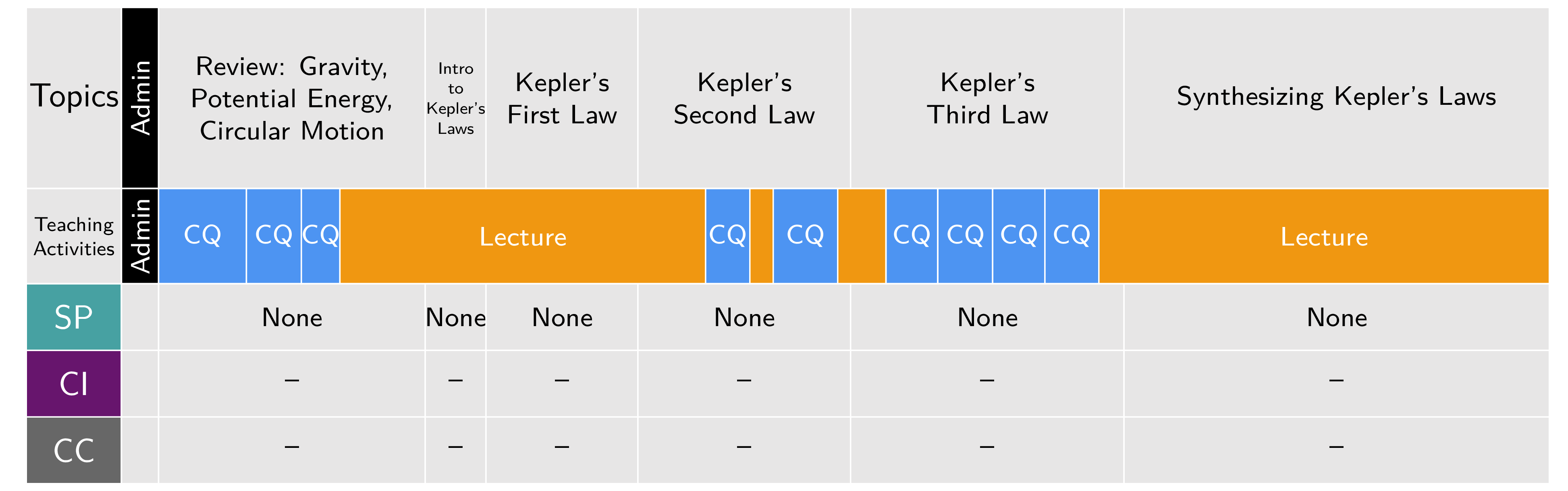

Supplement: S20 Fig — Introductory-Level Calculus-Based General Physics I. (TIFF) [file pone.0234640.s020.tiff]

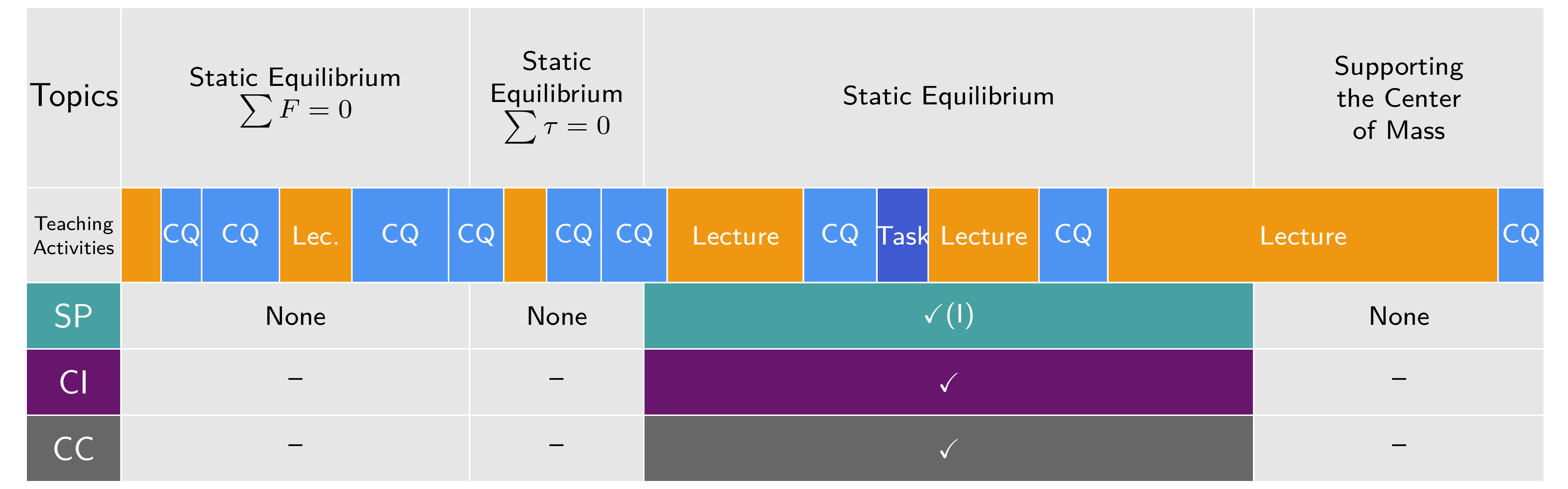

Supplement: S21 Fig — Introductory-Level Calculus-Based General Physics I. (TIFF) [file pone.0234640.s021.tiff]

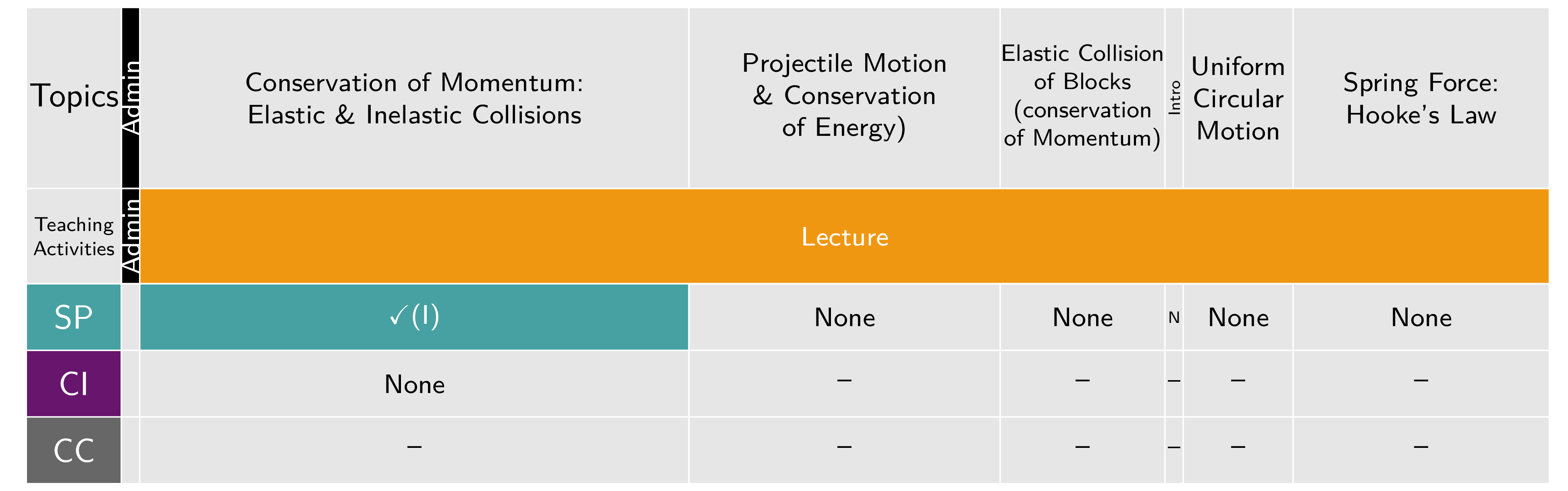

Supplement: S22 Fig — Introductory-Level Algebra-Based General Physics II. (TIFF) [file pone.0234640.s022.tiff]

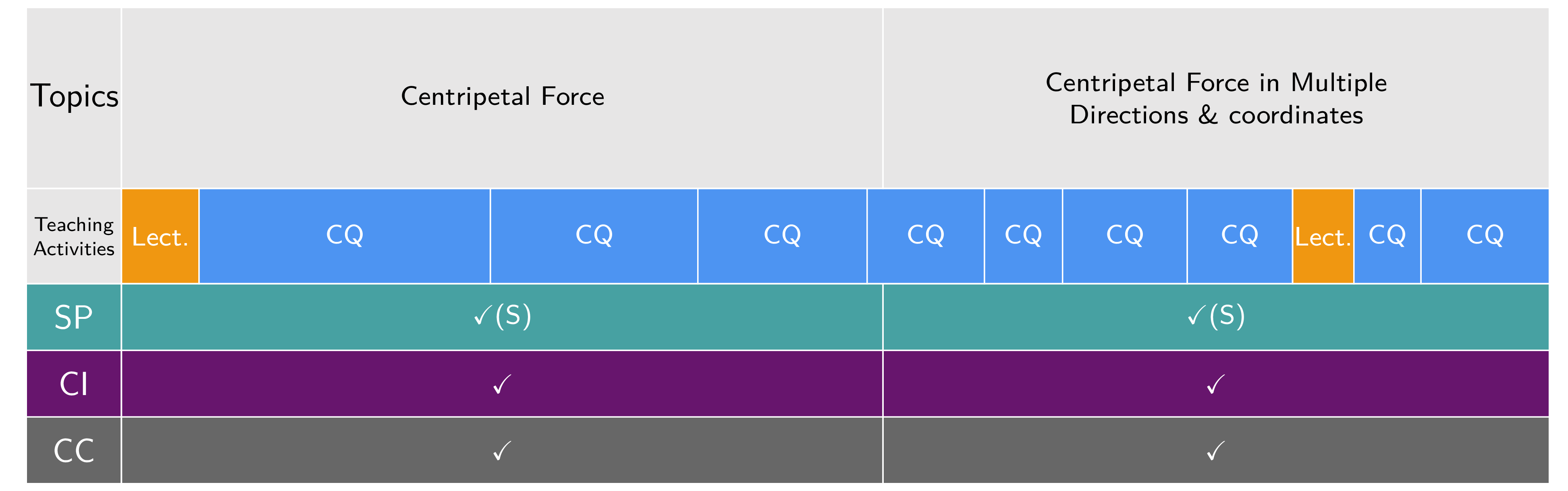

Supplement: S23 Fig — Introductory-Level Calculus-Based General Physics I. (TIFF) [file pone.0234640.s023.tiff]
