# Supplementary material for: Characterizing college science instruction: The Three-Dimensional Learning Observation Protocol
Source: PLoS One. 2020 Jun 16;15(6):e0234640. doi: 10.1371/journal.pone.0234640 (PMC7297354; doi:10.1371/journal.pone.0234640)
Supplement: S1 File — This document includes an expanded description of the 3D-LOP protocol. (DOCX) [file pone.0234640.s024.docx]

**Characterizing College Science Instruction: The Three-Dimensional Learning Observation Protocol**

Kinsey Bain, Rebecca L. Matz, Cori L. Fata-Hartley, Marcos D. Caballero, Diane Ebert-May, Sonia M. Underwood, Justin H. Carmel, Deborah G. Herrington, James T. Laverty, Erin M. Duffy, Jon R. Stoltzfus, Lydia Bender, Lynmarie A. Posey, Mark Urban-Lurain, Ryan L. Stowe, Ryan D. Sweeder, Stuart H. Tessmer, Melanie M. Cooper

**Supporting Information: 3D-LOP Protocol**

# Purpose

The Three-Dimensional Learning Observation Protocol (3D-LOP) is a protocol designed for two purposes:

1. To characterize instruction as having the potential for engagement with the three dimensions identified in *A Framework for K-12 Science Education: Practices, Crosscutting Concepts, and Core Ideas*.
2. To help instructors develop or modify existing instructional materials so that they have the potential for engagement with the three dimensions.

To accomplish these goals, the 3D-LOP describes criteria that can be used to determine if instruction has potential for engagement with the three dimensions. Because the 3D-LOP was developed primarily for college-level instruction, it was necessary to modify some aspects of the three dimensions outlined in the Framework. The 3D-LOP is a companion to the Three-Dimensional Learning Assessment Protocol (3D-LAP), in which a few of the scientific practices and crosscutting concepts were modified and new sets of core ideas were developed to be appropriate for college-level biology, chemistry, and physics courses.

## Characterizing Teaching Activities

While the primary motivation for developing the 3D-LOP was to characterize the content, it is still important to know how the class is being facilitated. The protocol for coding teaching activities was originally published in the supplemental materials of Matz et al. (13) and is included below with permission.

We coded the video data for the following six teaching activities, each of which are more fully described below:

- Clicker questions
- Tasks
- Interactions
- Lecture
- Administration
- Miscellaneous

For the purposes of coding and ease of use of the protocol, we defined these activities to be mutually exclusive and to completely cover each class, that is, every block of time in each class meeting from start to finish was coded as one and only one teaching activity. In the limited situations where more than one teaching activity was applicable, we applied the code that is listed first.

The development of these teaching activities and their definitions was based on our own discussions and viewing of local videos as well as descriptions of teaching practices in the Classroom Observation Protocol for Undergraduate STEM (COPUS) (1), Teaching Dimensions Observation Protocol (TDOP) (2), and Reformed Teaching Observation Protocol (RTOP) (3).

From a pool of five coders, two were randomly assigned to code each video. When the agreement between coders was greater than or equal to 90%, one of their timelines was randomly chosen as the final timeline for that video. When the agreement between coders was less than 90%, the coders met to reconcile their disagreement and used that reconciled timeline as the final one for that video.

Transitions between teaching activities were generally marked by one or more of:

- A change in slides
- A change in tone (e.g., verbal cues such as “So,” “Ok,” “Moving on,” “Now we’re going to look at,” and “Any questions?”)
- A change in content (e.g., moving from a specific instance to a generalizable principle; for a clicker question or task, moving the class away from answering or explaining the clicker question or task)

Following are the definitions and edge cases we described to facilitate reliably coding the teaching activities.

### *Clicker Questions*

Clicker questions are multiple-choice questions asked of students who respond with personal response instruments. The questions can be prepared or spontaneously generated.

The time recorded for a clicker question includes:

- The introduction to the clicker question
  - If the question is being spontaneously generated, the time to generate the question would be recorded.
- The clicker question itself
  - The presentation of the question itself.
  - The time that students actually use to work on the question, both working alone and in groups. This time is still coded as clicker question even if the instructor is walking around the room interacting with students.
- The conclusion of the clicker question
  - The time devoted to all report out dialogue and any brief follow-up explanation provided by the instructor. The follow-up might in practice be lecture (i.e., only the instructor is talking), a demonstration, or something else.

If multiple clicker questions are given back-to-back, then each new question should be marked separately. If the instructor polls the students, then lets the students think alone or discuss in groups, and then re-polls the students using the same question, the two questions should be marked separately.

If an instructor starts a clicker question before class so that students have the opportunity to click in before class starts, then the clicker question instance should start only once the instructor has begun to address the clicker question in class.

Sometimes, a clicker question instance will be complete, but then something, such as a student question, will prompt the class to go back and revisit that clicker question. Going back to a previous clicker question should be marked as a new clicker question instance.

During the clicker question, instructors may take a step back from the particulars of the question to discuss a relevant, more general concept. If this persists for a lengthy session of time, this should be coded as lecture or interaction as applicable. If the instructor returns to how this more general concept applies to the specific clicker question, then this should again be coded as clicker question. Another way to think about this is if the portion of time where the instructor takes a step back could be moved to before the clicker question started and still make sense, this should be coded as lecture or interaction as applicable. A transition to a new concept is likely a signal that the clicker question has ended.

If a task is assigned and the students work through it, but then the instructor follows that up with a clicker question where the instructor is asking for the answer they determined as part of the task, the whole time should be coded as a clicker question.

If a question is intended to be a clicker question (i.e., the instructor has the clicker options on the screen), but then the instructor collects the student responses via show of hands or shouting out the answer, this time should still be coded a clicker question.

Nonexamples of clicker questions:

- The instructor asks students for a quick “show of hands.”
- Extended follow-up lecture about the question or answer choices.

###

### *Tasks*

Tasks are items assigned to students to work on individually or in groups during class, however, clicker questions are recorded separately from tasks. Tasks can be prepared or spontaneously generated.

The time recorded for a task includes:

- The introduction to the task
  - The time that the instructor uses to introduce the task, explain any relevant directions and context, hand out microphones or materials, etc. Explaining the directions and context for the task might take a few slides and/or minutes.
- The task itself
  - The time that students actually use to work on the task, both working alone and in groups. This time is still coded as task even if the instructor is walking around the room interacting with students.
- The conclusion of the task
  - The time devoted to any report out dialogue and showing of student work (e.g., on a projector or through a short presentation to the entire class) during or after the students have completed the task. Similarly, this might take a few slides and/or minutes.

The goal in identifying the beginning and end of tasks, as for clicker questions, is to create “whole units,” meaning that if someone were to go back and look at the video recording of any task instance, they would see a logical lead up to the task, the task itself, and any brief conclusion to the task.

If multiple tasks are given back-to-back, each new task should be marked separately. Similarly, if the class session involves a very long task (such as a simulation), each subset of the task should be marked separately.

Just as for clicker questions, sometimes a task instance will be completed, but then something, such as a student question, will prompt the class to go back and revisit that task. Going back to a previous task should be marked as a new task instance.

If the report out for a task involves the instructor using multiple student responses as options for a clicker question, then the time should be marked as clicker question beginning with the visual generation of the clicker question options.

Just as for clicker questions, during the task, instructors may take a step back from the particulars of the task to discuss a relevant, more general concept. If this persists for a lengthy session of time, this should be coded as lecture or interaction as applicable. If the instructor returns to how this more general concept applies to the specific task, then this should again be coded as task. Another way to think about this is if the portion of time where the instructor takes a step back could be moved to before the task started and still make sense, this should be coded as lecture or interaction as applicable. A transition to a new concept is likely a signal that the task has ended.

Examples of tasks:

- Students engage in think/pair/share questions.
- Students are asked to think about something on their own and then at least one student reports their idea to the class.
- Students create diagrams.
- Students draw on paper or whiteboards.

### *Interactions*

Interactions are substantive and potentially lengthy exchanges between the instructor and one or more students about the content (not logistics) of the course. To qualify as an interaction, it is required that “student responses are either guiding or being integrated within the discussion” [citation]. “Student responses” includes but is not limited to in-class verbal responses from students as well as out-of-class responses to homework or exam questions. “Guiding or being integrated” requires that the student responses are important in the trajectory of the conversation.

Interactions include when a student raises a question or makes a comment to which the instructor or another student responds. The attention of most of the class is focused on this interaction, and the instructor might present materials intermittently throughout the interaction.

An easy way to identify an interaction is if the instructor and students stay on the same topic for a substantive session of time, but this is not a necessary condition.

Examples of interactions:

- The instructor uses student homework answers to facilitate a discussion.
- The instructor talks to the students while asking multiple, successive questions to which the students respond. The student responses are guiding or being integrated within the discussion.

Nonexamples of interactions:

- Questions about administration or logistics. (This should be coded as administration.)
- Students talk to one another in groups. (This should be coded as task.)
- The instructor asks any number of short “call-and-response” types of questions (especially with little to no wait time). (This should be coded as lecture.)
- The instructor asks rhetorical questions, which they may or may not answer themselves. (This should be coded as lecture.)
- The instructor invites one or more students to help with a demonstration.

Note that any of the above nonexamples of interactions could occur *within* an interaction instance in which an authentic interaction is happening. On their own, however, these nonexamples do not warrant marking interaction.

### *Lecture*

Lecture is instructor-directed presentation of content-related information, including learning objectives.

Examples of lecture:

- PowerPoint presentations.
- Writing and drawing on chalkboards, whiteboards, overheads, and document projectors.
- Presentation of animations and videos.
- Content-related instruction about what will happen in future recitations, labs, class meetings, exams, etc. For example, saying, “Here is a list of topics that will be covered on the next exam” would be coded as lecture, not administration, because it is content-related.

### *Administration*

Administration is when instructors go over “housekeeping items” such as logistics, scheduling, and announcements.

By definition, administration does not focus on course content. Discussions about metacognition or learning styles are coded as clicker questions, tasks, interactions, or lecture as appropriate.

Examples of administration:

- Assigning homework.
- Discussing logistics for an upcoming exam.
- Discussing grade results of a homework assignment or exam.

### *Miscellaneous*

All class time that cannot be coded as one of the teaching practices above should be coded as miscellaneous. All class time that is coded as miscellaneous should be described in a free-form text label associated with the instance.

Examples of miscellaneous:

- An informational presentation from or about an outside group, such as an advertisement.
- Disruptions such as a cell phone ringing that draw the attention of the class and last for, in general, one minute or more.
- Delays due to technical difficulties (e.g., changing batteries) or otherwise.

## Characterizing Instruction

The 3D-LOP criteria are applied to instruction as enacted by the instructor. That is, the 3D-LOP criteria target instructional opportunities for the instructor and students to engage with each of the dimensions and does not attempt to determine how students are responding to curricular materials (e.g., off task conversations in small groups).

Unlike in the 3D-LAP where the unit of analysis is clearly defined (e.g., a single question or a cluster of questions), the unit of instruction is less obvious. To help focus analysis by reducing cognitive demand when coding and increase resolution beyond a whole class session (without coding too frequently, e.g., every two minutes, which was found to be unfruitful), videos are portioned into “segments”. A segment is a continuous segment of a class session that has a coherent set of content focused around the same topic(s). Multiple types of classroom activities can be part of the same segment (e.g., lecture and clicker questions) if they are related by topic. It is also common that instructor cues can be used to help identify segments (e.g., using phrases such as “reviewing from the previous class session” or “moving on to a new topic”). The segments from an example biology class session are shown in Fig S1.


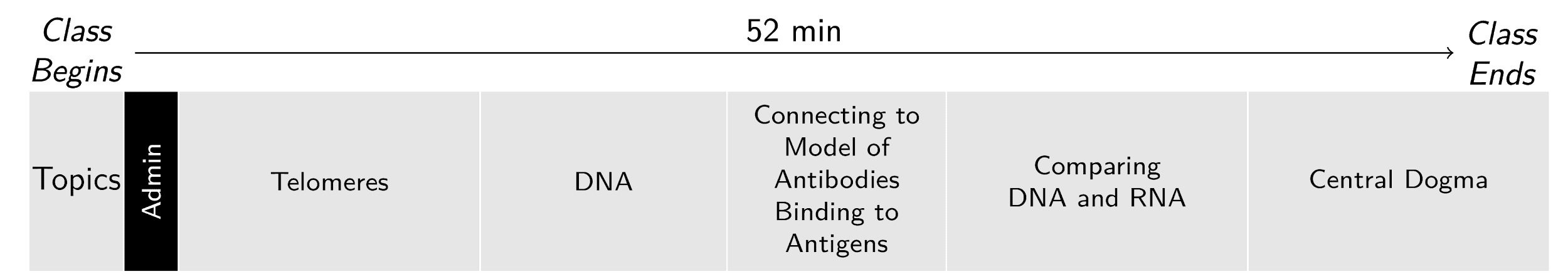


**Fig S1. Example of the segments in a biology class session video.**

During the development of the 3D-LOP, videos were divided into segments according to topic by a single disciplinary expert in biology, chemistry, or physics from our research team. In rare cases when they were uncertain, they would seek input from another member of the research team. Each segment was later coded by two disciplinary experts from our team using the dimension criteria (the two coders were usually different from the original segmenter). During the coding process, the coders were able to adjust the segmenting if deemed necessary after further review of the video and the segmenting guidelines.

When coding for the dimensions, the unit of instruction is a “segment” of the class session. Once a video is segmented, each segment may be subsequently coded using the dimension criteria for scientific practices, core ideas, and crosscutting concepts. Many of the classroom observations contained segments that were administrative in nature (e.g., announcements, reminders, or grade updates). These administrative segments were not coded using the dimension criteria. The dimension coding for the same example biology recording is shown in Fig S2. This class session began with an administrative segment; therefore, it was not coded using the dimension criteria.


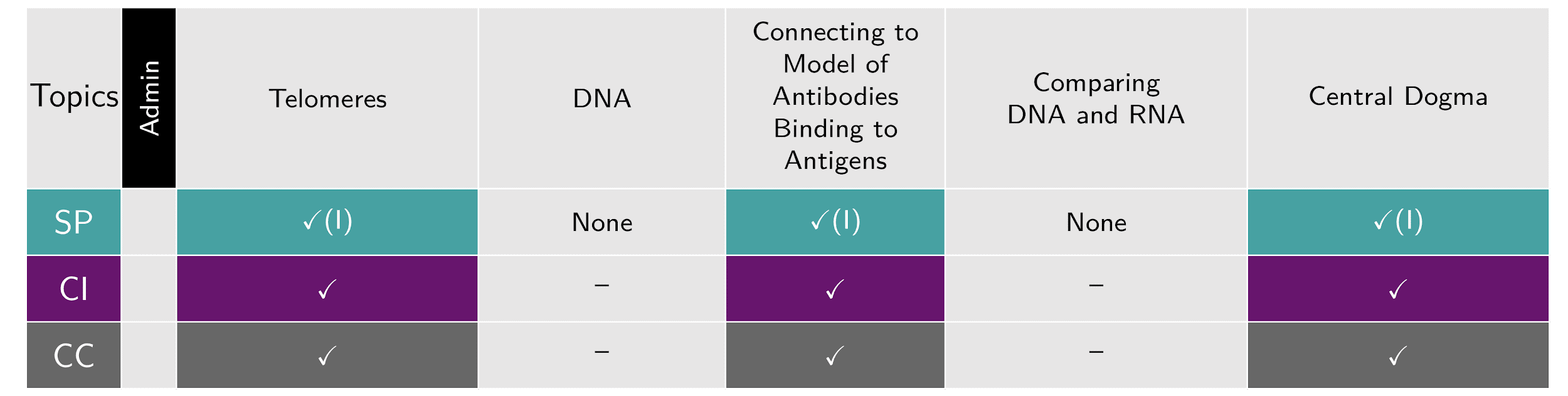


**Fig S2.** **Example of the dimension coding for each segment in a biology class session video.**

Each class session was also coded for teaching activities. Because the protocol for teaching activity coding is independent of the segments, the codes from teaching activity analysis do not necessarily map neatly onto each segment (though they often can). Using the same biology class session, Fig S3 shows the teaching activity coding (note that in this video many of the segments start and stop at the same time point as a teaching activity code).


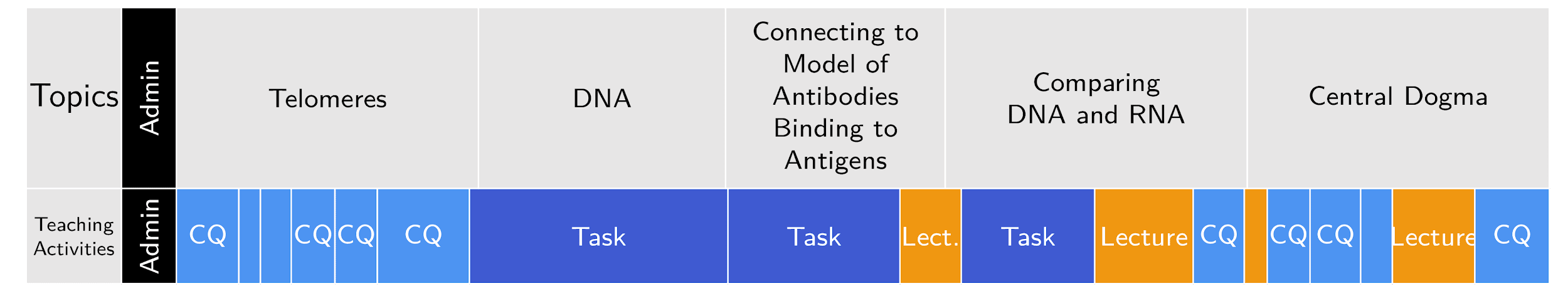


**Fig S3.** **Example of the teaching activity coding in a biology class session video.**

Compiling the segmenting and coding for teaching activities and the dimensions provides rich insight to a given class session. An example of a possible representation, a compiled timeline, for the biology class session (Figs S1-S3) can be seen in Fig S4. This timeline output reveals the topics addressed during instruction (segmenting), how the class was facilitated (teaching activity coding), and engagement with the dimensions (dimension coding). It can be used to characterize the extent to which the instructors and students are engaging with each of the three dimensions, as well as how this is being facilitated.


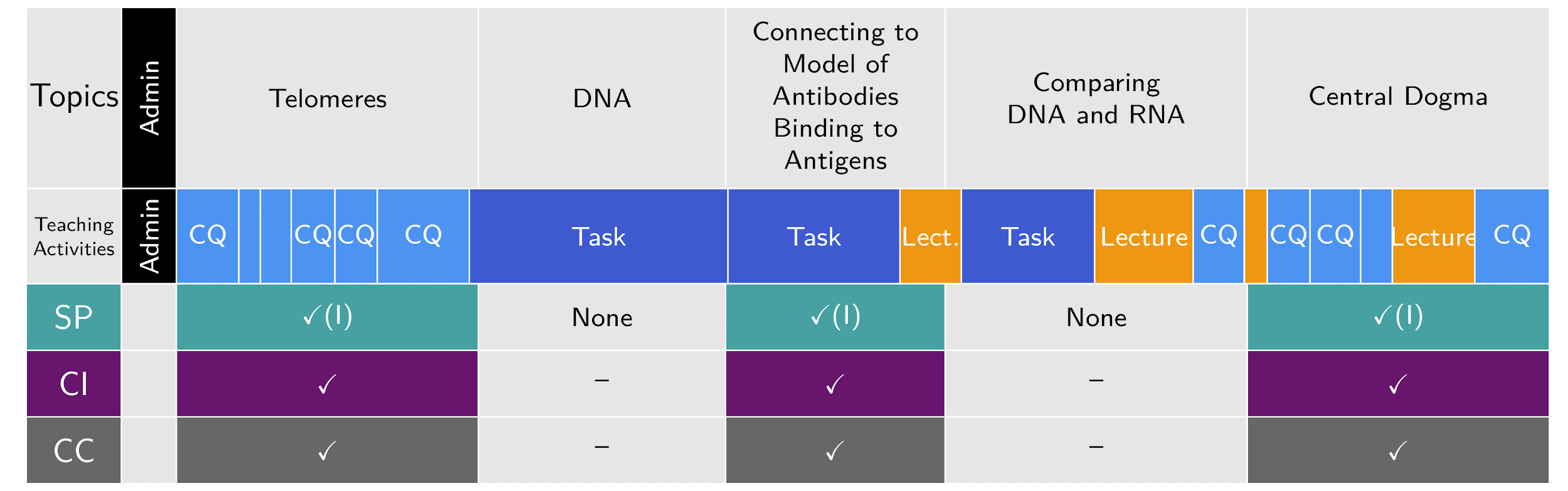


**Fig S4.** **Compiled 3D-LOP timeline of segmenting with coding for teaching activities and dimensions in a biology class session video.**

## The 3D-LOP Dimension Criteria

To characterize each segment in a video, a coder must identify if the segment meets the criteria for a given dimension. Unlike the 3D-LAP, the 3D-LOP criteria are sequential in that there is an order of operations when it comes to coding each of the dimensions (whereas the dimensions were more independent in the 3D-LAP criteria). Fig S5 depicts the process of coding using the 3D-LOP dimension criteria, where each segment of a video is reviewed by a coder. The coder first identifies if the segment meets all of the criteria for one of the scientific practices. If yes, the coder also identifies who is primarily engaging in the scientific practice (instructor or student). The coder then identifies if the segment reflects any of the listed core ideas for that discipline. If it does, the coder then goes to the final stage of identifying if the segment meets the criteria for a crosscutting concept. If a segment meets the criteria for each of the dimensions, it is considered to be “3D”. However, if at any point in the coding process, a segment does not meet the criteria for one of the dimensions, the coding stops, and the segment is not considered to be “3D”.


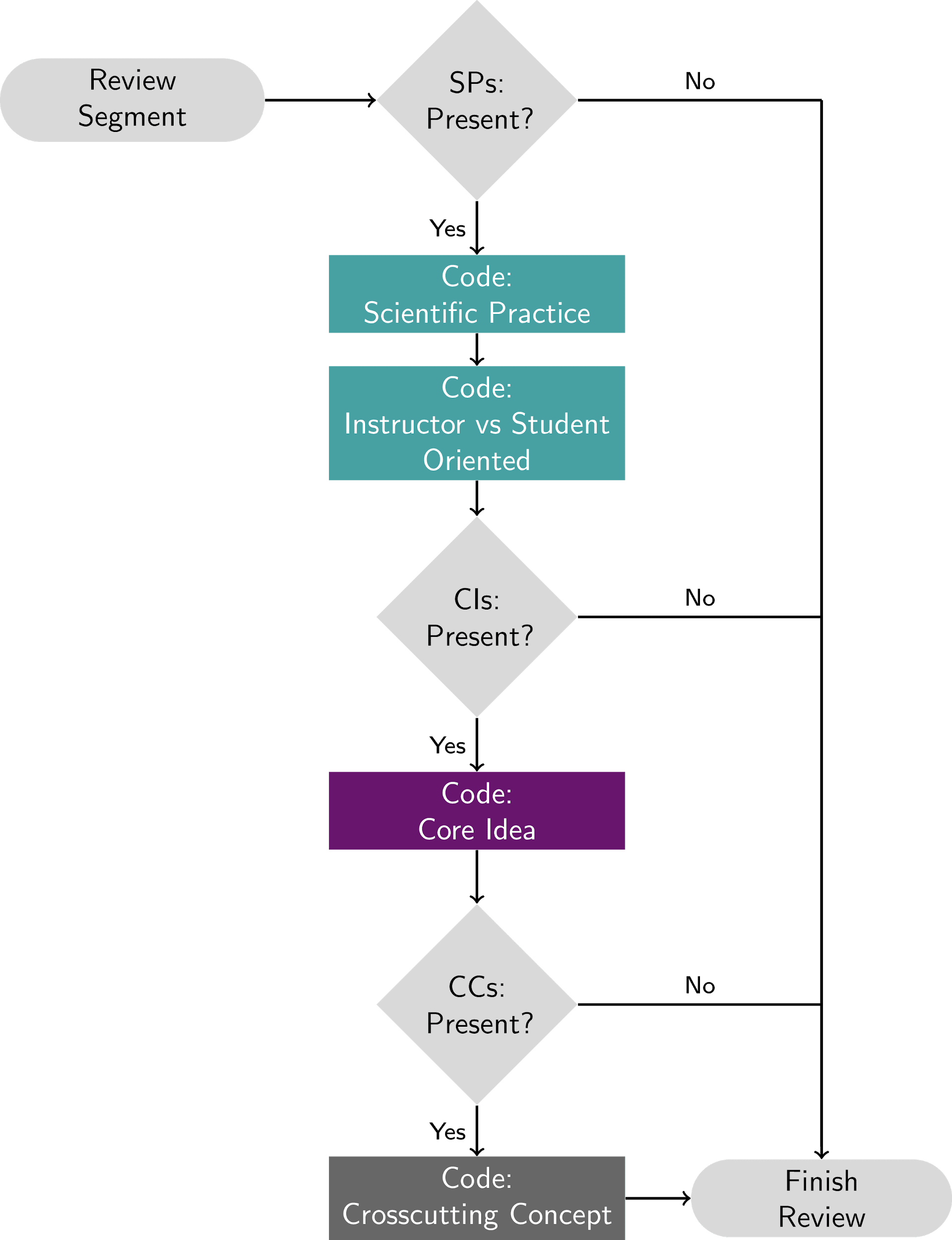


**Fig S5. Flowchart to guide the coding of a segment using the 3D-LOP dimension criteria, where if the answer to the question in each arrow is “no”, stop coding the segment.**

The criteria for each of the dimensions was adapted from the 3D-LAP criteria. A full detailed description of the criteria development for the 3D-LAP can be found in Laverty et al. [citation]. Unlike the 3D-LAP, the 3D-LOP only has one set of criteria for engagement with the scientific practices (different sets of criteria were needed to characterize constructed-response and selected response assessment items). Due to the varying nature of classroom engagement, a set of criteria were developed for the 3D-LOP to characterize *who* was doing most of the engagement with scientific practices (instructor or student).

## Constructing or Revising Instructional Materials

There are a number of ways to design instructional materials that have the potential to engage students with three-dimensional learning. For example, instructors might choose a scientific practice, crosscutting concept, and core idea that they wish to target, using the criteria in the 3D-LOP to guide their writing of instructional materials. Alternatively, instructors might examine existing materials and think about how to modify them so that they have the potential to engage students in three-dimensional learning.

## Scientific Practices Criteria

### Asking Questions

1. Instruction presents an event, observation, phenomenon, data, scenario, or model.
2. Instruction has instructor/students *generate* an empirically testable question about the given event, observation, phenomenon, data, scenario, or model. (Question cannot be rhetorical or simply a part of instruction; rather it must be a *process of generating* a testable question.)

### Developing and Using Models

1. Instruction presents an event, observation, or phenomenon for instructor/students to explain or make a prediction about.
2. Instruction presents a representation or asks instructor/students to construct a representation.
3. Instruction has instructor/students explain or make a prediction about the event, observation, or phenomenon.
4. Instruction has instructor/students provide the reasoning that links the representation to their explanation or prediction.

### Planning Investigations

1. Instruction poses a scientific question, claim, or hypothesis to be investigated.
2. Instruction has instructor/students describe or design an investigation, or identify the observations required to answer the question or test the claim or hypothesis.
3. Instruction has instructor/students justify how their description, design, or observations can be used to answer the question or test the claim or hypothesis.

### Analyzing and Interpreting Data

1. Instruction presents a scientific question, claim, or hypothesis to be investigated.
2. Instruction provides a representation of data (table, graph, or list of observations) used to answer the question or test the claim or hypothesis.
3. Instruction provides an analysis of the data or asks instructor/students to analyze the data.
4. Instruction has instructor/students interpret the results or assess the validity of the conclusions in the context of the scientific question, claim, or hypothesis.

### Using Mathematics and Computational Thinking

1. Instruction presents an event, observation, or phenomenon.
2. Instruction has instructor/students perform a calculation or statistical test, generate a mathematical representation, or demonstrate a relationship between parameters.
3. Instruction has instructor/students give a consequence or an interpretation in words, diagrams, symbols, or graphs of their mathematical results in the context of the given event, observation, or phenomenon.

### Constructing Explanations and Engaging in Argument from Evidence

1. Instruction presents an event, observation, or phenomenon.
2. Instruction presents or asks instructor/students to make a claim based on the given event, observation, or phenomenon.
3. Instruction has instructor/students provide scientific principles or evidence (data or observations) to support the claim.
4. Instruction has instructor/students provide reasoning about why the scientific principles or evidence support the claim.

### Evaluating Information

1. Instruction provides an excerpt from a conversation, article, student solution, or other communication that makes one or more assertions.
2. Instruction presents a conclusion about the validity of the assertion(s) made or asks instructor/students to make a conclusion about the validity of the assertion(s), or reconcile multiple assertions with each other.
3. Instruction has instructor/students provide reasoning to support their conclusion(s) about the validity of the assertion(s) or reconciliation with data, observations, or scientific principles.

## Instructor vs. Student Engagement in Scientific Practices Criteria

Instructor vs. student engagement is coded with respect to the scientific practices. When coding this category, a helpful consideration is to think about each scientific practice criteria bullet point from the perspective of who is doing what.

## Instructor-Oriented Scientific Practice Engagement: This can range from instructor-only engagement in the scientific practice (no questions, does not matter that a student is in the classroom) to when students are limited to very short responses (instructors expecting quick, essentially one-word responses from students, this is a floor for clicker questions, not a ceiling).

## Student-Oriented Scientific Practice Engagement: This can range from instructors expecting longer than one word answers from students (that is, there is at least some potential for students to engage in pieces of the scientific practice, but instructors are doing much of the intellectual work) to scaffolded intellectual exchange between student and instructors to primarily student-oriented engagement in the scientific practice with spurious instructor input and guidance (potential for rich student engagement).

If a segment has more than one scientific practice with different instructor/student engagement levels, the *highest* level of student engagement is coded (e.g. if one SP in the segment is instructor-oriented, but another SP is student-oriented, code this segment as student-oriented).

# Core Ideas

## Biology Core Ideas

1. **Chemical and Physical Basis of Life**: Life processes are the result of regulated chemical and physical interactions and reactions governed by the laws of physics.
2. **Matter and Energy**: Free energy and matter are used in regulated processes that establish order, support growth and development, and control dynamic homeostasis.
   - Thermodynamically favorable reactions are coupled with thermodynamically unfavorable reactions in these regulated molecular processes.
   - Molecules and atoms from the environment are rearranged to build new molecules in these regulated molecular processes.
   - Photosynthesis, cellular respiration, and trophic dynamics are key processes mediating the cycling of matter and flow of energy at multiple scales (from cells to ecosystems).
3. **Cellular Basis of Life**: Cells are the fundamental units of all living things.
   - Cells are created from other cells.
   - Cells interact with other cells.
4. **Systems**: Ecosystems, organisms, tissues, and cells act as systems.
   - Dynamic molecular interactions result in the emergence of increasingly complex biological properties.
   - Changes in chemical and physical signals affect the structure and performance of cell systems.
   - Organisms interact with their abiotic and biotic environments at multiple scales for the purpose of obtaining resources. These interactions mediate movements of matter, energy, and information in ecological systems and are subject to physical and chemical laws.
     - The distribution and abundance of organisms -- as well as the direction, magnitude, and frequencies of their interactions -- can change in space and time and are linked to availabilities of matter, energy, and other resources.
   - Evolutionary histories, random/stochastic effects, and humans impact ecosystem structure, function, and dynamics.

1. **Structure and Function**: The functions and properties of ecosystems, organisms, tissues, cells, and biological molecules are determined by their structures.
   - At the molecular level, biology is based on dynamic, three-dimensional chemical and physical interactions.
2. **Information Flow, Exchange, and Storage**: Hereditary information is stored, used, and replicated.
   - DNA is the source of heritable information in a cell.
   - The growth and behavior of organisms is determined by the information contained in their genes and by the regulated expression of those genes.
     - The molecular structure and function of cells is regulated by gene expression.
     - Gene expression and protein activity are regulated by intracellular and extracellular signaling molecules that vary over time and depend on environmental conditions.
   - The transmission of DNA between generations follows predictable patterns.
     - Meiosis produces gametes for sexual reproduction.
     - Mendelian genetics predicts many patterns of inheritance.
     - Additional inheritance patterns are related to the assortment of linked genes, epigenetic changes, and the vertical and horizontal transfer of genetic information.
3. **Evolution:** Evolution drives the diversity and unity of life.
   - Evolution is the change in allele and/or genotypic frequencies in a population and is manifested in the changes in inherited characteristics over generations.
   - Evolutionary change is driven by natural selection and genetic drift. Variation, caused by random mutation and passed on by reproduction, provides the raw material for evolution.
   - Organisms are linked by lines of descent from common ancestry. Phylogenetic trees are hypotheses of the pattern of descent from a common ancestor which can be explored and tested.
   - Evolution affects the genetic makeup of populations over time and can lead to adaptation, speciation, and extinction.
   - Evolution can be tested using the Hardy-Weinberg Equilibrium as the null hypothesis.

## Chemistry Core Ideas

1. **Electrostatic and Bonding Interactions**: Attractive and repulsive electrostatic forces govern noncovalent and bonding (covalent and ionic) interactions between atoms and molecules. The strength of these forces depends on the magnitude of the charges involved and the distances between them.
   - Attractive noncovalent interactions (intermolecular forces) between atoms and molecules arise from interactions between transient, induced, and permanent dipoles.
   - Atoms also interact through electrostatic forces to form chemical bonds, which have greater stability (lower energy) than the separated atoms. Nonpolar covalent bonding and ionic bonding represent the limits in a continuum of bonding interactions with polar covalent bonds falling in-between. Electrons in the highest energy orbitals with the largest spatial extent (valence electrons) are used to form bonds.
   - Covalent and ionic bonding interactions are typically stronger than noncovalent (intermolecular) interactions.
2. **Atomic/Molecular Structure and Properties**: The macroscopic physical and chemical properties of a substance are determined by the three-dimensional structure, the distribution of electron density, and the nature and extent of the noncovalent interactions between particles.
   - The three-dimensional structure of a molecule is determined by the atoms that make up the molecule, the repulsions between the bonding and nonbonding electrons, and the distribution of the valence electrons within the molecule.
   - The properties of a molecule are determined by the interactions and associated energy and entropy changes that occur when molecules interact.
3. **Energy**: Energy changes are either the cause or the consequence of change in chemical systems, which can be considered on different scales and can be accounted for by conservation of the total energy of the system of interest and the surroundings.
   - Macroscopic: Changes in phase and reactions of collections of atoms and/or molecules are accompanied by energy changes that result from energy changes on the atomic/molecular scale.
     - Typically, these energy changes are observed by a change in temperature or a phase change.
     - Temperature is determined by the average kinetic energy of collections of atoms and/or molecules.
   - Atomic/Molecular: Kinetic and potential energy changes occur when atoms and molecules interact. Energy is released to the surroundings when bonds or attractive noncovalent interactions form, and conversely energy is required to break bonds or noncovalent interactions.
     - The overall energy change that accompanies a chemical reaction is the net result of the energy added to break bonds and the energy released when new bonds form.
     - Energetic barriers along the path between reactants and products (reaction coordinate) determine the rates of reaction.
   - Quantum Mechanical Energy Levels and Changes: Energy levels are quantized in atoms and molecules resulting in discrete energies for transitions between energy levels. This is a direct consequence of the wave-particle duality of electrons and other subatomic particles.
     - Energy levels are quantized in atoms and molecules.
     - Evidence for the quantization of energy levels comes from spectroscopy. The features of observed spectra correspond to transitions between quantized energy states and directly provide the energy difference between initial and final energy states.
     - The electromagnetic radiation that interacts with atoms and molecules in spectroscopic measurements also exhibits wave-particle duality.
4. **Change and Stability in Chemical Systems**: Energy and entropy changes, the rates of competing processes, and the balance between opposing forces govern the fate of chemical systems.
   - Change: Change in chemical systems results from the natural evolution of the system or occurs in response to a perturbation to the system.
   - Stability: Stability in a chemical system results from the balancing of forces or the rates of competing processes. While we often ascribe a move towards “stability” as the driving force for a particular change in a system, these underlying ideas are the mechanism by which stability is achieved.
   - Gibbs Energy and Entropy: The thermodynamic function Gibbs free energy captures the roles of enthalpy and entropy in determining the direction of change of a chemical system. The Gibbs free energy is a proxy for the total entropy change of the universe (system + surrounding) based on properties of the system.

## Physics Core Ideas

1. **Interactions Can Cause Changes in Motion**: Changes in an object’s motion are the result of interactions between it and one or more other objects. Multiple interactions between an object and its surroundings can result in a predictable change in motion.
   - All macroscopic forces are the result of gravitational or electromagnetic interactions between particles.
   - Non-zero net forces cause changes in linear momentum.
   - Non-zero net torques cause changes in angular momentum.
   - Changes in linear and angular momentum can be used to predict the motion of objects.
2. **Energy is Conserved**: Energy comes in many forms and can be transformed from one form to another within a given system or transferred between systems.
   - ΔE = W + Q; the total energy of a system changes based on a system’s interactions with its surroundings. These interactions can be separated into the work done on/by the surroundings (W) and the thermal energy exchanged with the surroundings (Q).
   - Work is due to macroscopic interactions between objects.
   - Thermal energy exchanged with the surroundings is due to molecular level interactions between constituent particles.
3. **Exchanges of Energy increase Total Entropy**: Multiparticle systems tend toward states that are more statistically likely to occur. At a macroscopic scale, this can be described by concepts such as entropy, temperature, and pressure.
   - Energy is quantized.
   - Energy can be distributed within a system of particles in many different ways (microstates).
   - Left alone, systems tend towards macrostates with a larger number of associated microstates.
   - The total entropy of the universe is always increasing.
   - The total energy change of a system at a given temperature is related to the mechanical work done (pressure-volume work) on/by the system and the change in entropy of that system at the given temperature.
4. **Interactions are Mediated by Fields**: Fields are generated by charges/masses. Fields affect charges/masses. In circuits, fields induce currents.
   - All charges generate electric fields.
   - Moving charges generate magnetic fields.
   - Net electric fields affect all charges.
   - Net magnetic fields affect moving charges.
   - Masses generate gravitational fields.
   - Net gravitational fields affect all masses.
5. **Energy, Momentum, Angular Momentum, and Information can be Transported without a Net Transfer of Matter**: Mechanical waves move through matter. Electromagnetic waves can move through vacuum or matter. Properties of waves can be used to parameterize the information or amount of energy, momentum, or angular momentum is transported.
   - Mechanical waves cause matter to oscillate in order to transfer energy, momentum, angular momentum, and information without a net transfer of material.
   - Electromagnetic radiation exhibits both wave and particle properties, and these models can be used to investigate or explain different phenomena.

# Crosscutting Concepts Criteria

## Patterns

Instruction identifies patterns or trends emerging from three or more events, observations, or data.

## Cause and Effect: Mechanism and Explanation

Instruction provides at most two of the following and the instructor or student identifies the other(s) (must be all 3):

1. a cause
2. an effect
3. the mechanism that links the cause to the effect

## Scale, Proportion, and Quantity

### Scale

Instruction (must be at least one):

1. compares objects, processes, or properties across size, time, or energy scales, or to dimensions of familiar objects, timescales, or energies.
2. identifies non-negligible/relevant interactions at various scales.

### Proportion and Quantity

Instruction (must be at least one):

1. predicts the response of one variable to changes in another variable.
2. identifies the relationship between two or more variables from data.

## Systems and System Models

Instruction (must be all 4):

1. identifies a system by defining its components or boundaries.
2. identifies any assumptions inherent in the model.
3. identifies the surroundings (if necessary).
4. discusses how the system and surroundings interact with each other.

## Energy and Matter: Flows, Cycles, and Conservation

Instruction (must be all 3):

1. describes the transfer or transformation of energy or matter *across* systems.
2. describes the transfer or transformation of energy or matter *between* a system and its surroundings.
3. recognizes/discusses *explicitly* that energy and/or matter are conserved.

## Structure and Function

Instruction (must be at least one):

1. predicts or explains a function or property based on structure.
2. describes what structure could lead to a given function or property.

## Stability and Change

Instruction (must be at least one):

1. determines if a system is stable and provide evidence for this.
2. determines what forces, rates, or processes make a system stable (static, dynamic, or steady state).
3. determines under what conditions a system remains stable.
4. determines under what conditions a system is destabilized and the resulting state.

# References

1. Smith MK, Jones FHM, Gilbert SL, Wieman CE. The classroom observation protocol for undergraduate STEM (COPUS): A new instrument to characterize university STEM classroom practices. CBE-Life Sci Educ. 2013 Dec 21;12(4):618–27.

2. Hora MT. Toward a Descriptive Science of Teaching: How the TDOP Illuminates the Multidimensional Nature of Active Learning in Postsecondary Classrooms. Sci Educ. 2015 Sep;99(5):783–818.

3. Sawada D, Piburn MD, Judson E, Turley J, Falconer K, Benford R, et al. Measuring reform practices in science and mathematics classrooms: The reformed teaching observation protocol. Sch Sci Math. 2002 Oct 1;102(6):245–53.
