# Supplementary material for: Characterizing college science instruction: The Three-Dimensional Learning Observation Protocol
Source: PLoS One. 2020 Jun 16;15(6):e0234640. doi: 10.1371/journal.pone.0234640 (PMC7297354; doi:10.1371/journal.pone.0234640)

**Characterizing College Science Instruction: The Three-Dimensional Learning Observation Protocol**

Kinsey Bain, Rebecca L. Matz, Cori L. Fata-Hartley, Marcos D. Caballero, Diane Ebert-May, Sonia M. Underwood, Justin H. Carmel, Deborah G. Herrington, James T. Laverty, Erin M. Duffy, Jon R. Stoltzfus, Lynmarie A. Posey, Mark Urban-Lurain, Ryan L. Stowe, Ryan D. Sweeder, Stuart H. Tessmer, Melanie M. Cooper

**Supporting Information: Exemplars**

This document contains timelines of class sessions from each discipline using video recordings captured before and after course transformations. Each class session timeline shows the segments, characterization of teaching activities, and coding from the 3D-LOP. The segmenting and coding criteria can be found in the Supporting Information: 3D-LOP.

## Biology Exemplars

**Fig S6. Traditional Biology Example 1:** Introductory-Level Cell and Molecular Biology Class Session


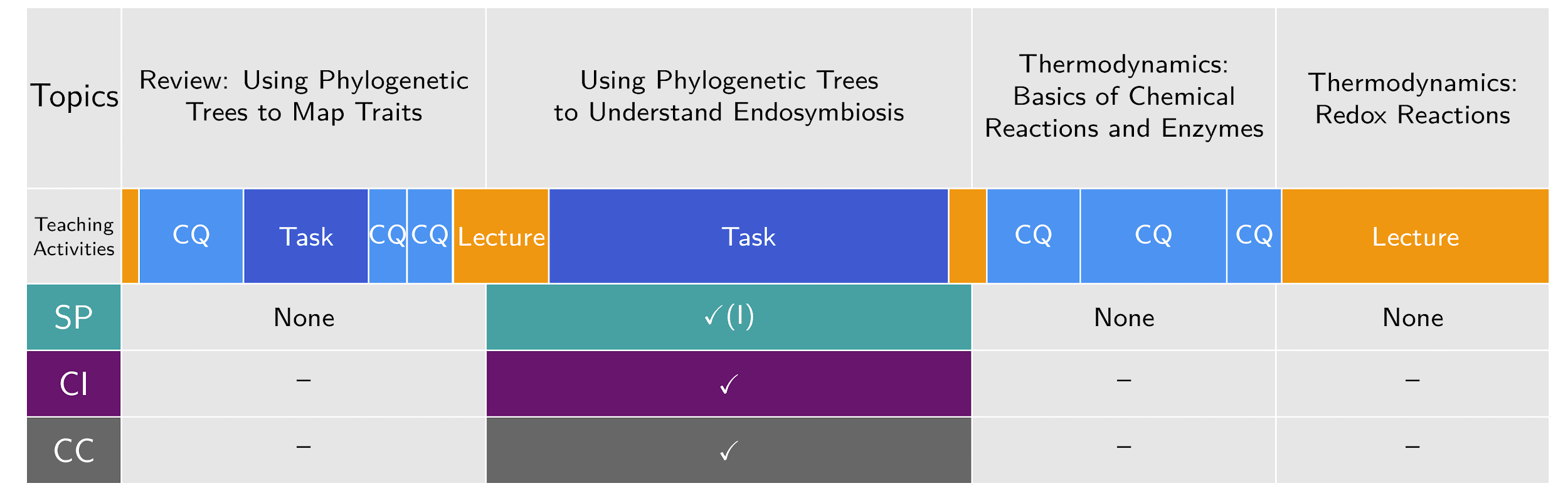


**Fig S7. Traditional Biology Example 2:** Introductory-Level Cell and Molecular Biology Class Session


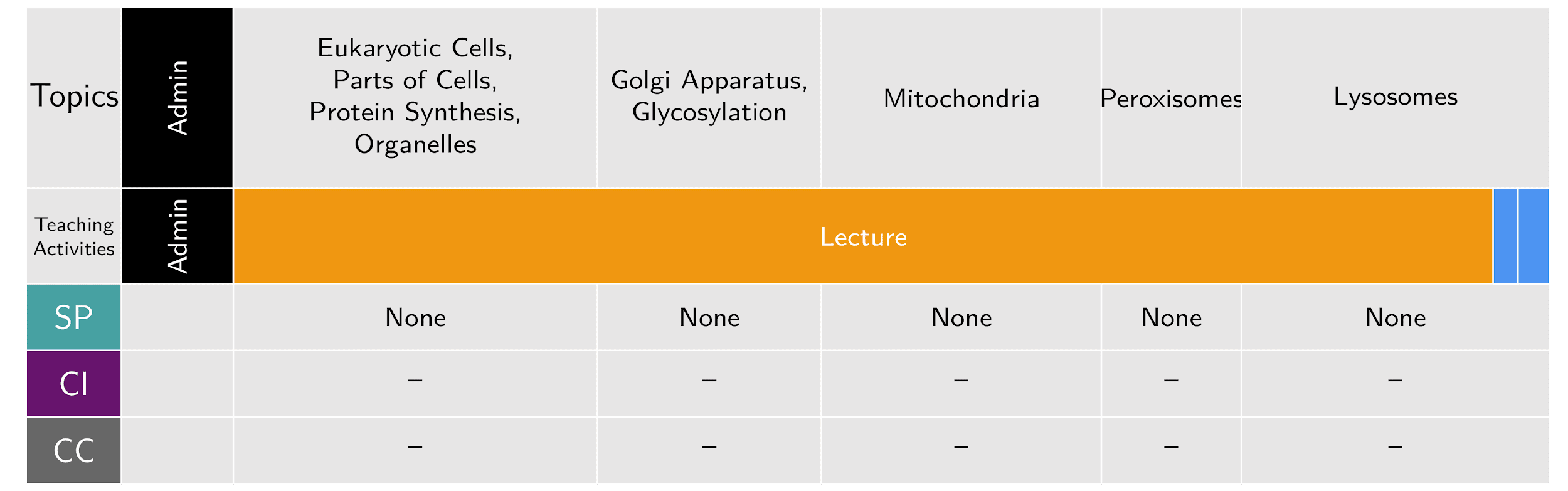


**Fig S8. Traditional Biology Example 3:** Introductory-Level Organismal and Population Biology Class Session


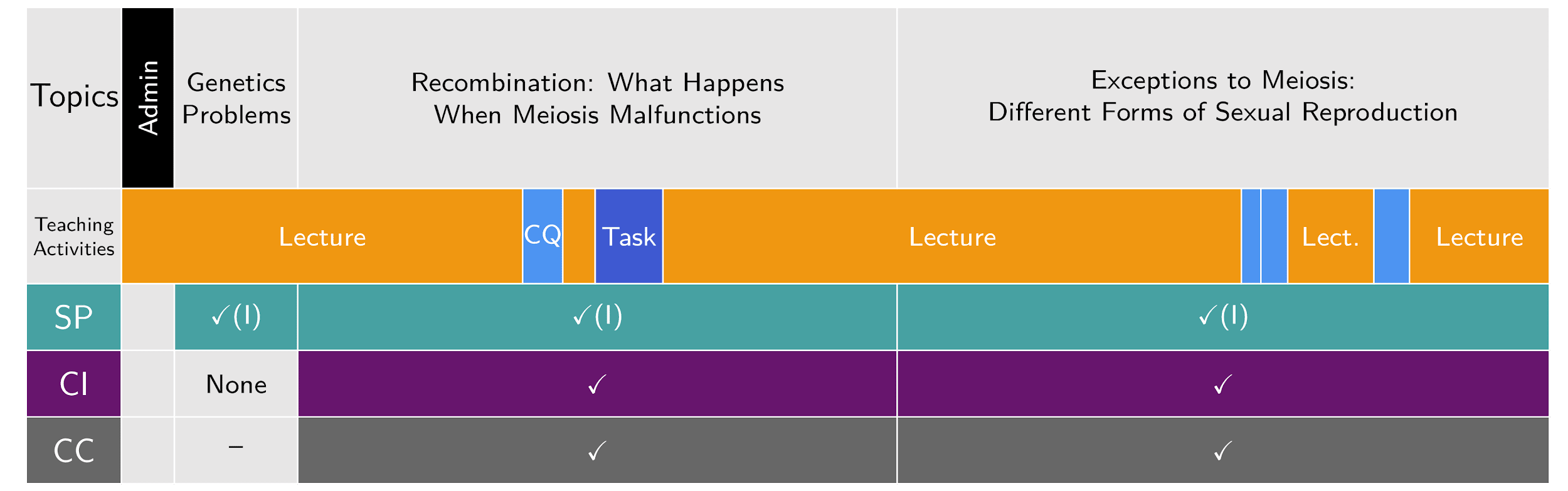


**Fig S9. Transformed Biology Example 1:** Introductory-Level Cell and Molecular Biology Class Session


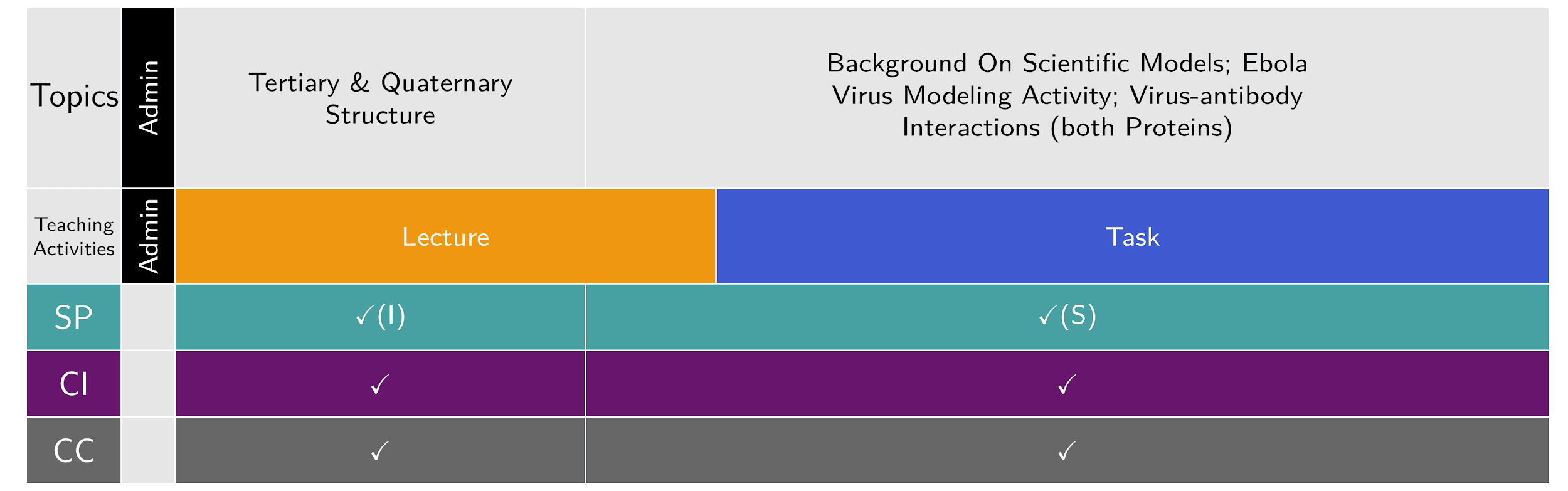


**Fig S10. Transformed Biology Example 2:** Introductory-Level Cell and Molecular Biology Class Session


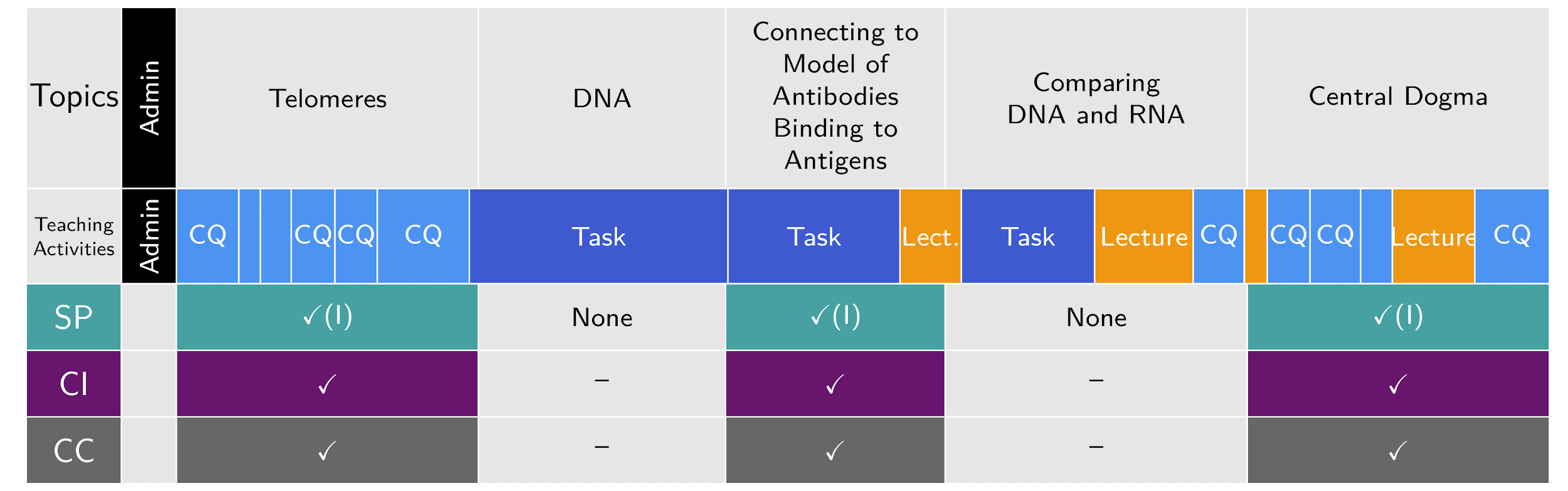


**Fig S11. Transformed Biology Example 3:** Introductory-Level Organismal and Population Biology Class Session


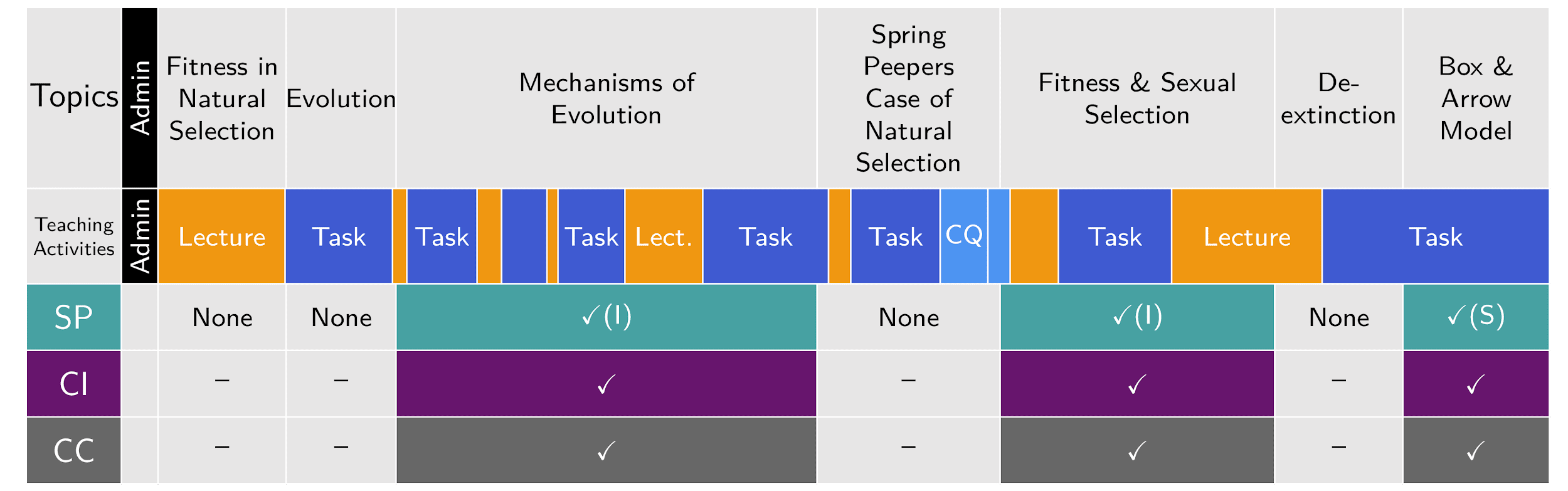


## Chemistry Exemplars

**Fig S12. Traditional Chemistry Example 1:** Introductory-Level General Chemistry II


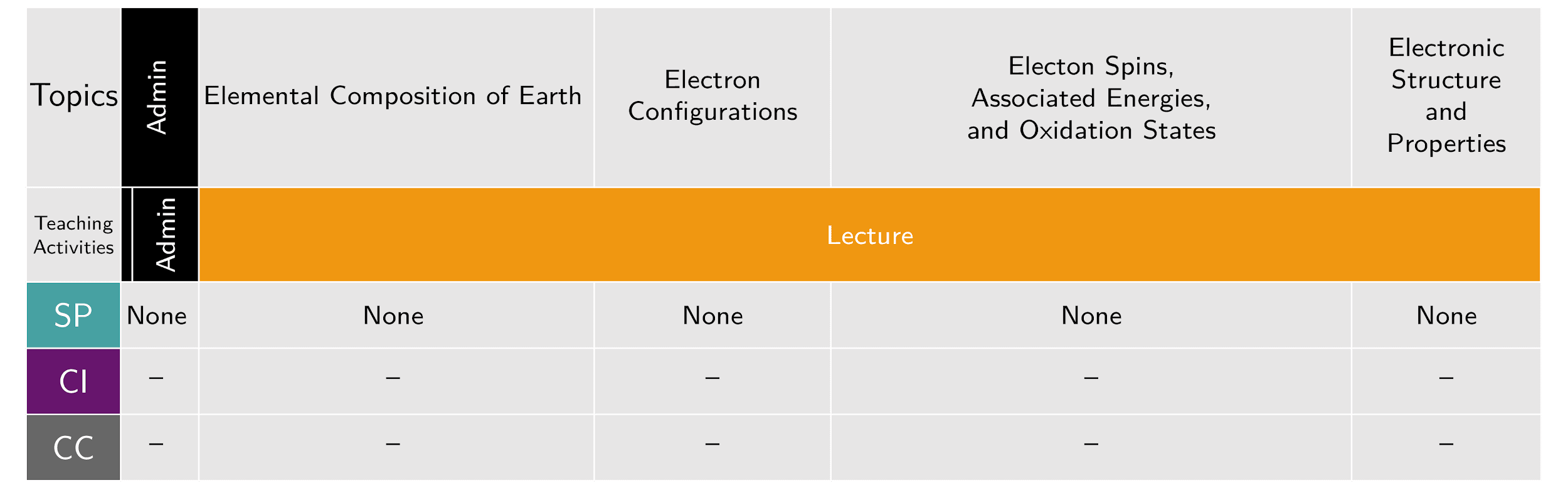


**Fig S13. Traditional Chemistry Example 2:** Introductory-Level General Chemistry I


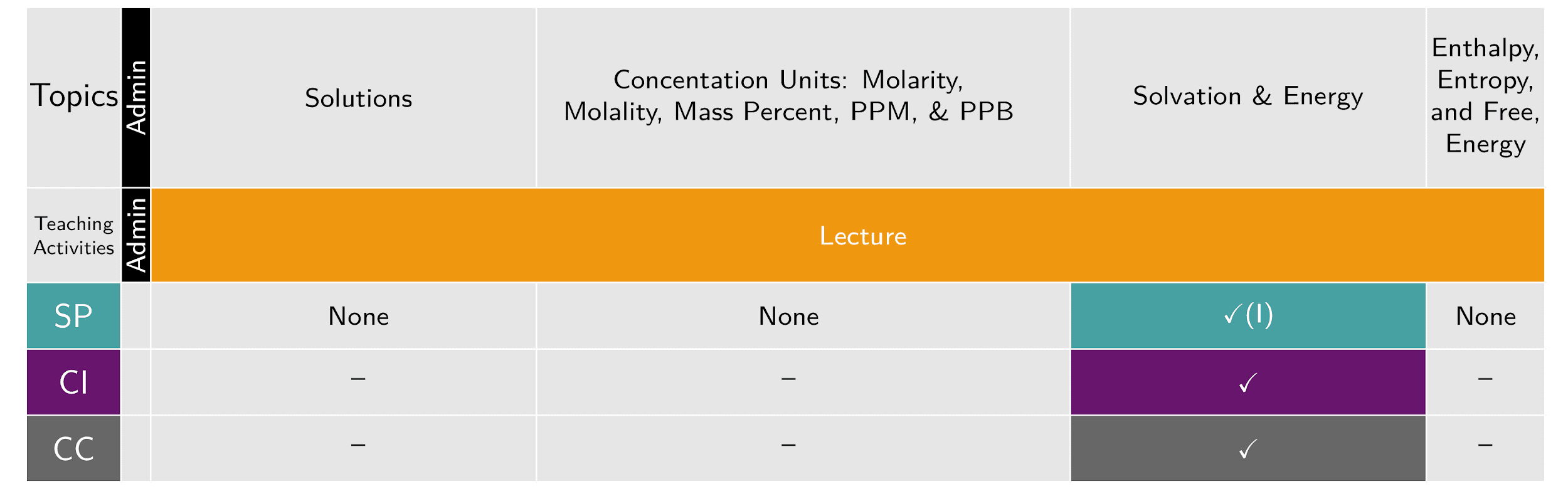


**Fig S14. Traditional Chemistry Example 3:** Introductory-Level General Chemistry I for Majors


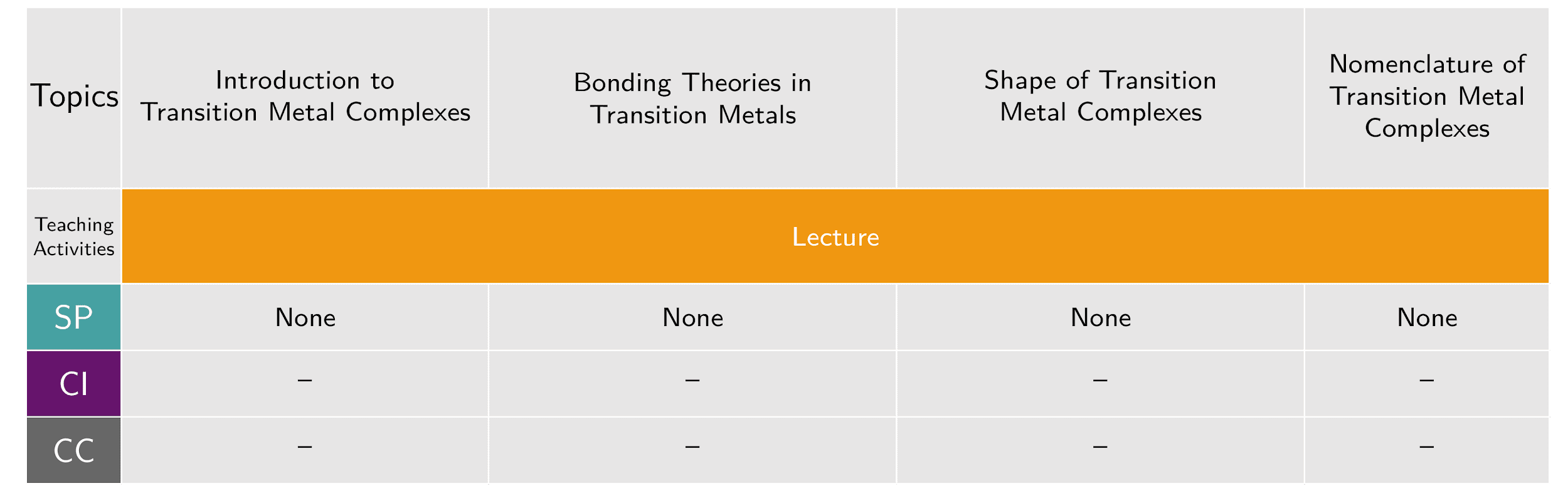


**Fig S15. Transformed Chemistry Example 1:** Introductory-Level General Chemistry II


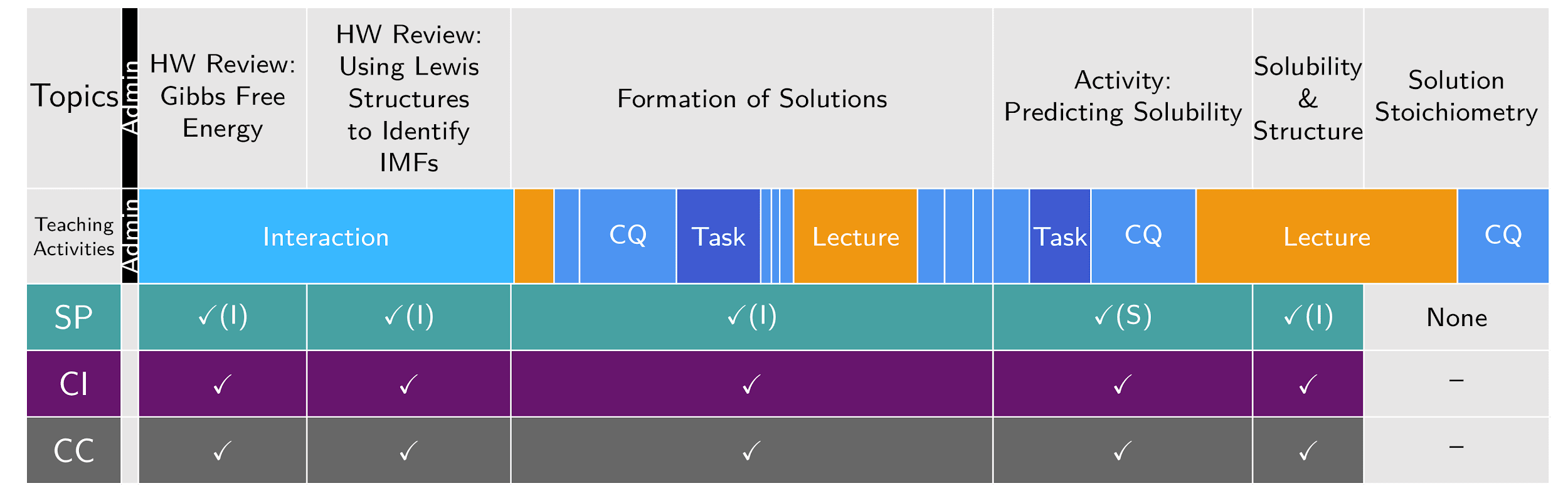


**Fig S16. Transformed Chemistry Example 2:** Introductory-Level General Chemistry II


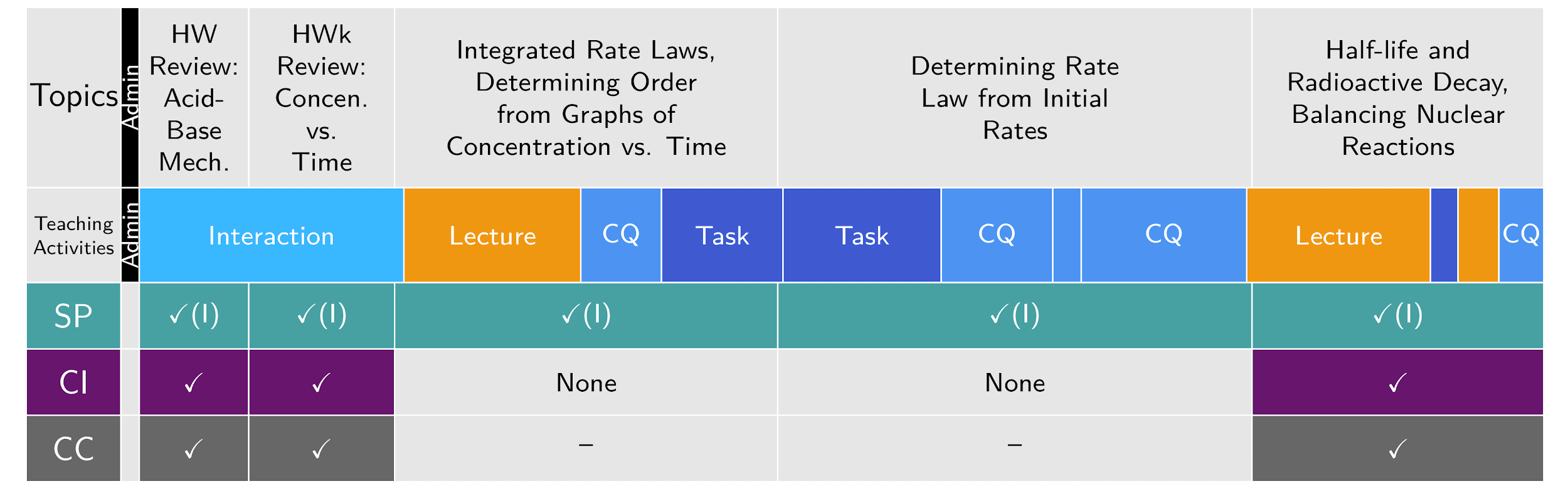


**Fig S17. Transformed Chemistry Example 3:** Introductory-Level General Chemistry I


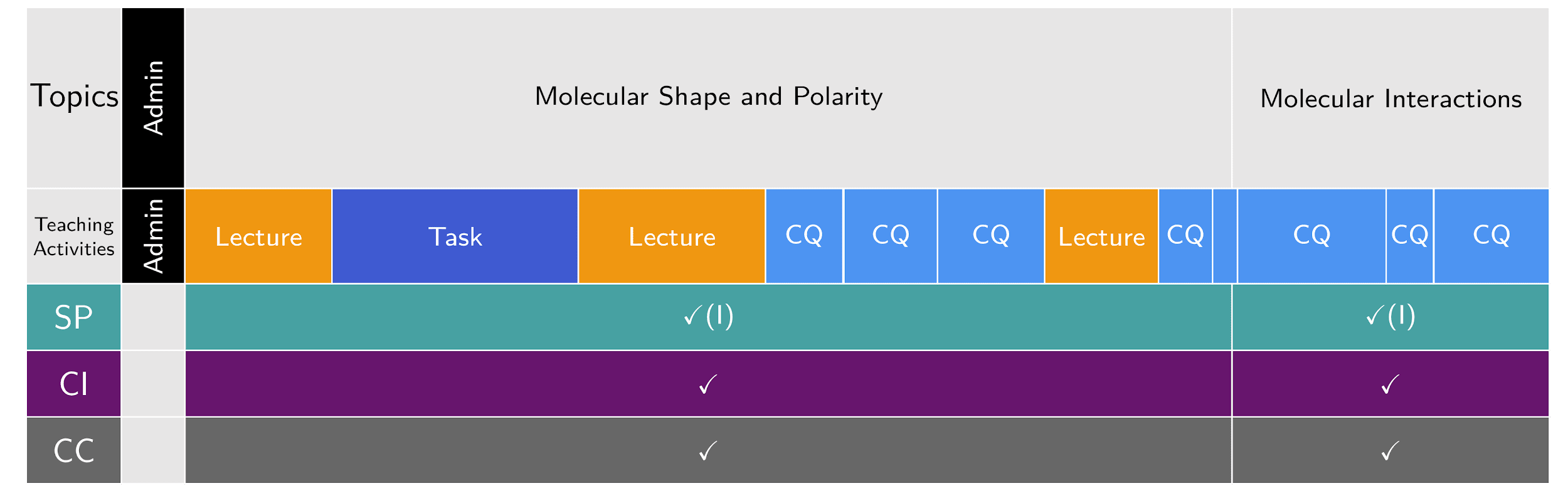


## Physics Exemplars

**Fig S18. Traditional Physics Example 1:** Introductory-Level Calculus-Based General Physics II


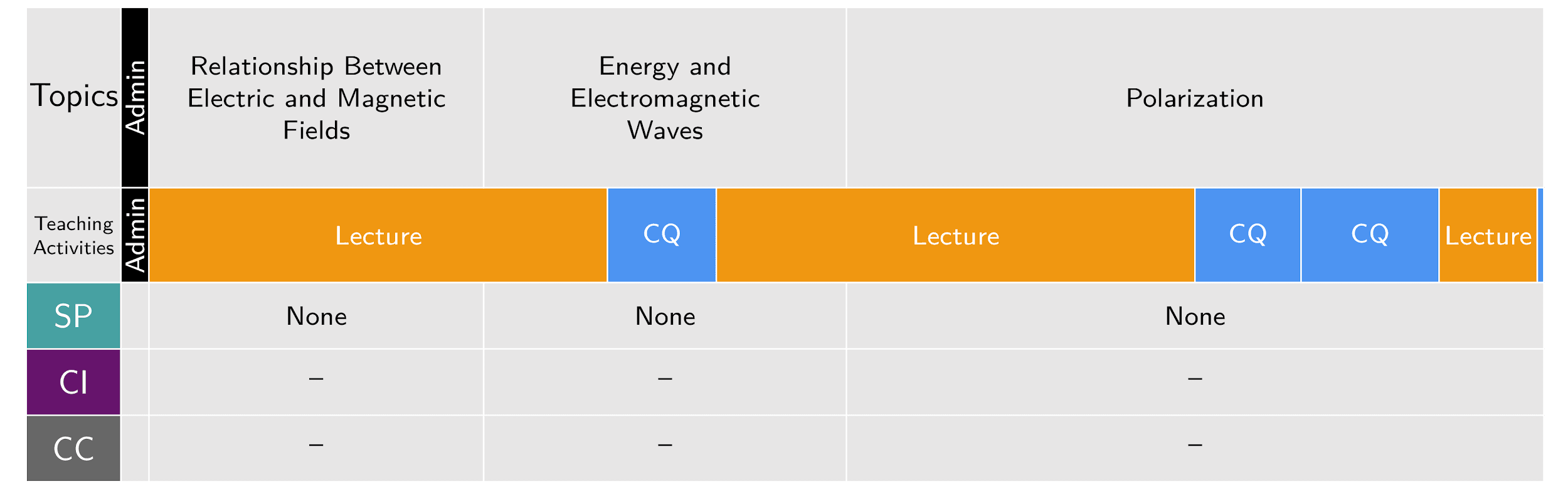


**Fig S19. Traditional Physics Example 2:** Introductory-Level Calculus-Based General Physics II


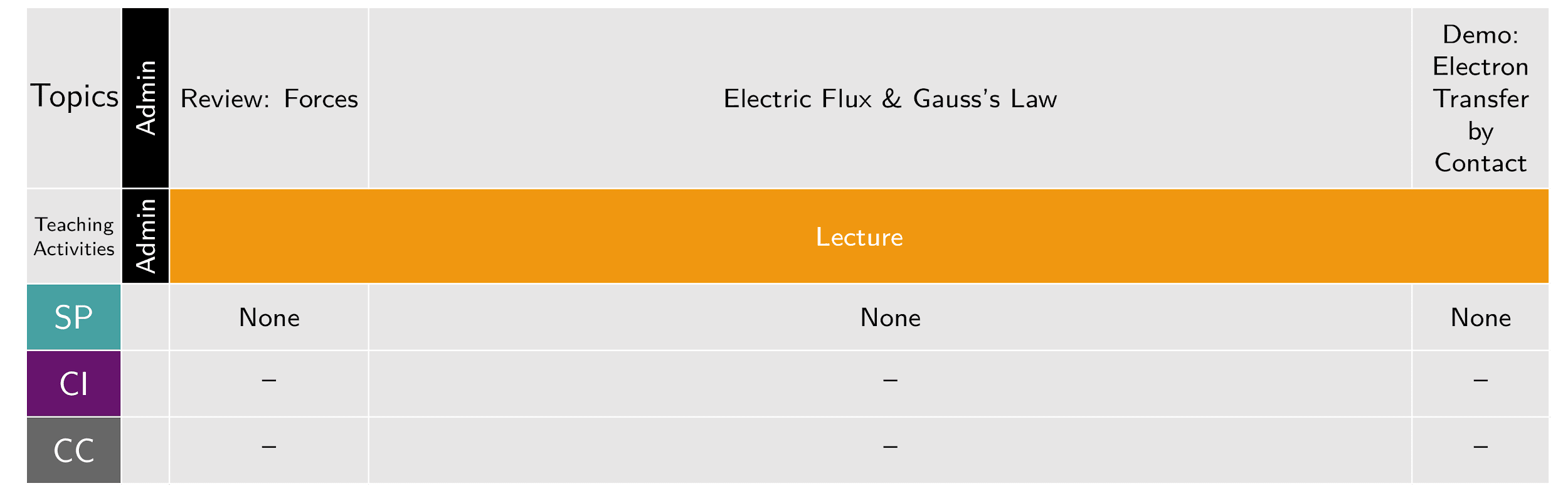


**Fig S20. Traditional Physics Example 3:** Introductory-Level Calculus-Based General Physics I


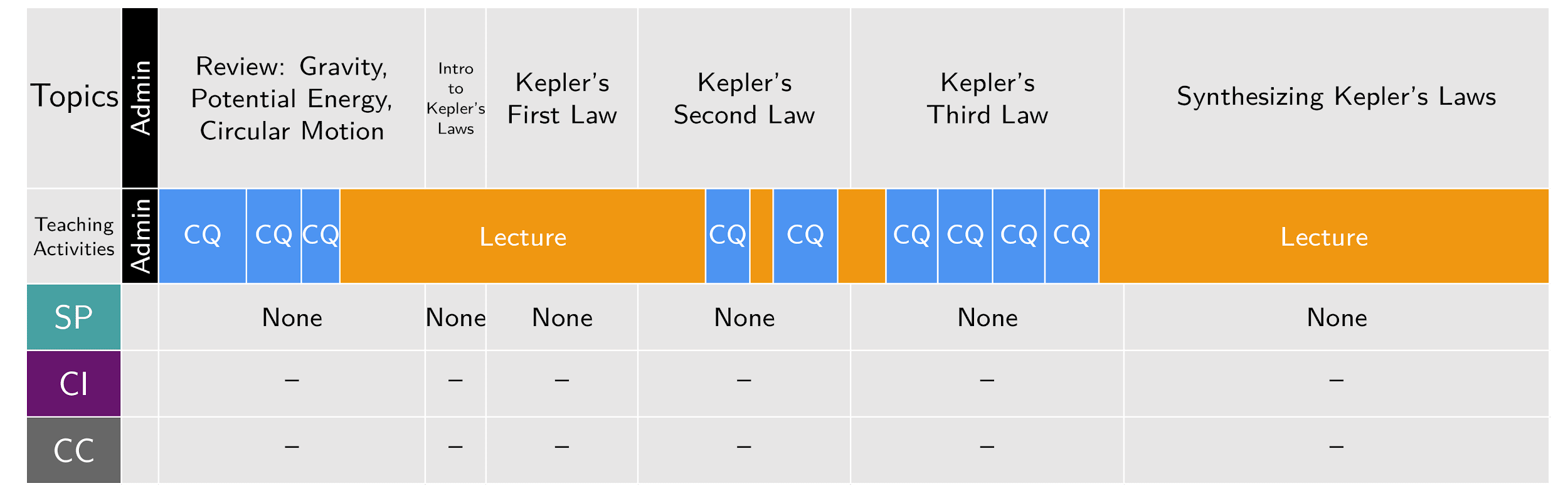


**Fig S21. Transformed Physics Example 1:** Introductory-Level Calculus-Based General Physics I


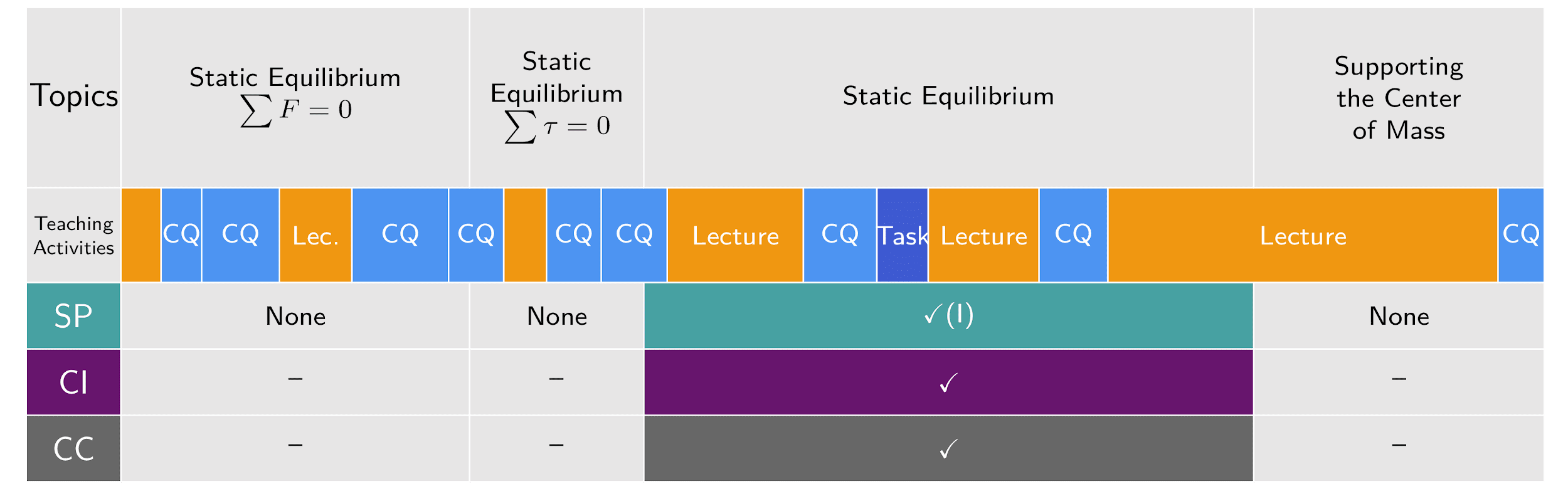


**Fig S22. Transformed Physics Example 2:** Introductory-Level Algebra-Based General Physics II


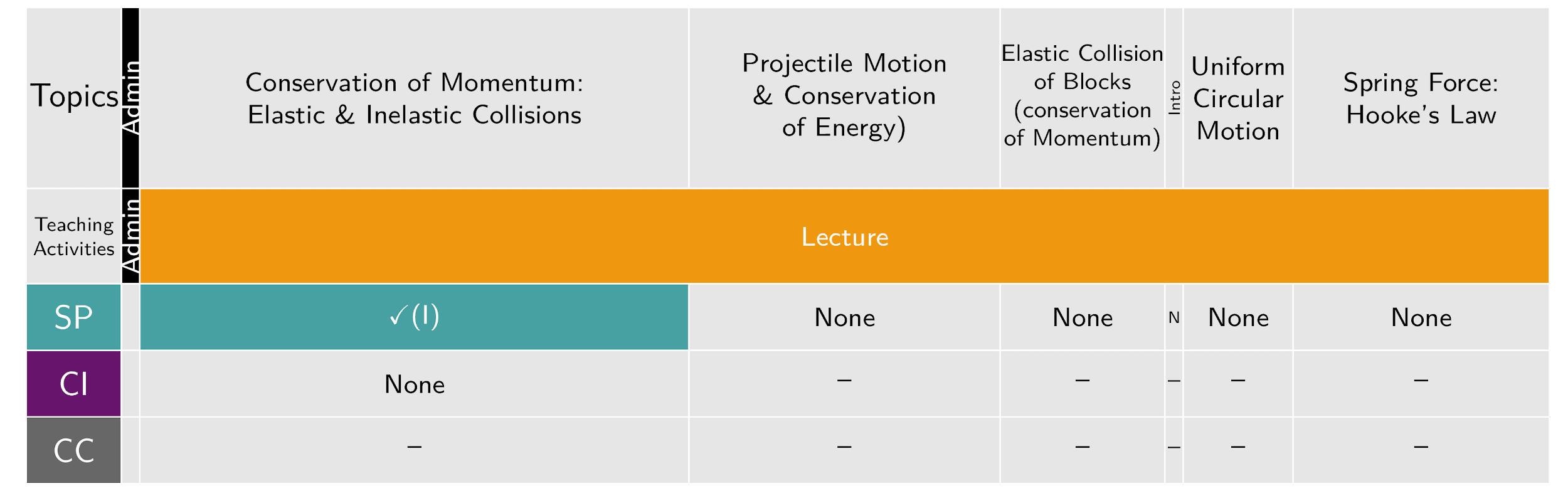


**Fig S23. Transformed Physics Example 3:** Introductory-Level Calculus-Based General Physics I


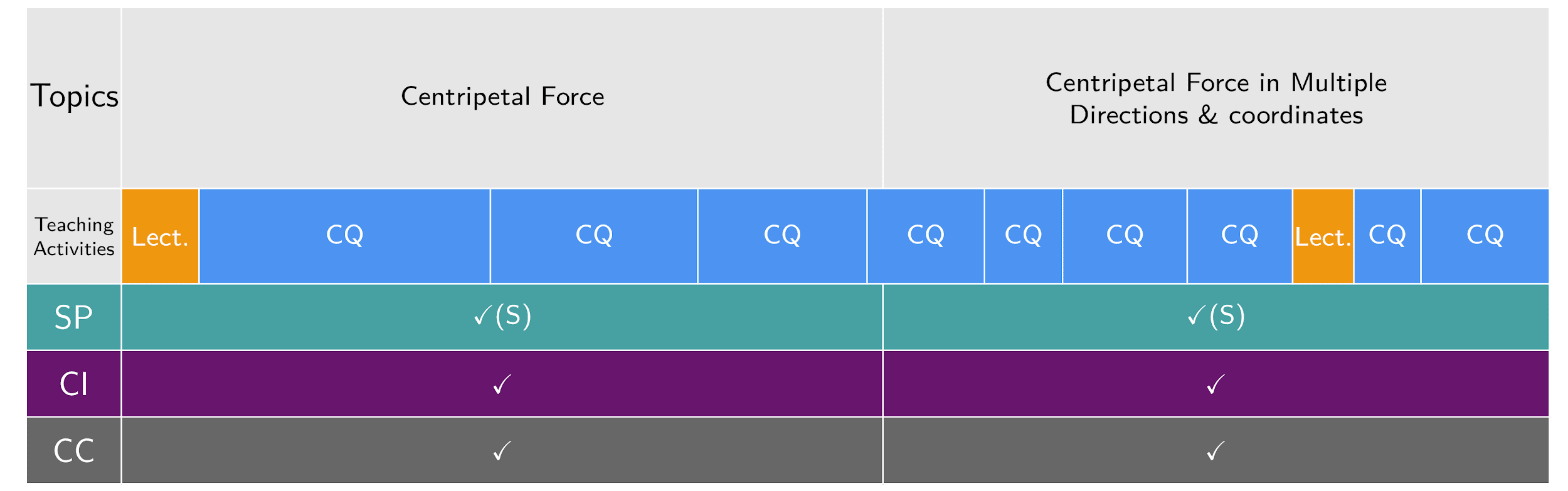

Supplement: S2 File — This document contains timelines of class sessions from each discipline using video recordings captured before and after course transformations. Each class session timeline shows the segments, characterization of teaching activities, and coding from the 3D-LOP. (DOCX) [file pone.0234640.s025.docx]
